# Fluorogenic Detection of Sulfite in Water by Using Copper(II) Azacyclam Complexes

**DOI:** 10.3390/molecules27061852

**Published:** 2022-03-12

**Authors:** Carlo Ciarrocchi, Donatella Sacchi, Massimo Boiocchi, Maduka Lankani Weththimuni, Alessio Orbelli Biroli, Maurizio Licchelli

**Affiliations:** 1 Dipartimento di Chimica, Università di Pavia, Via Taramelli 12, 27100 Pavia, Italy; carlo.ciarrocchi@unipv.it (C.C.); donatella.sacchi@unipv.it (D.S.); madukalankani.weththimuni@unipv.it (M.L.W.); alessio.orbellibiroli@unipv.it (A.O.B.); 2 Centro Grandi Strumenti, Università di Pavia, Via Bassi 21, 27100 Pavia, Italy; massimo.boiocchi@unipv.it

**Keywords:** copper complexes, macrocyclic compounds, azacyclam, fluorescent probes, sulfite detection

## Abstract

Copper(II) azacyclam complexes (azacyclam = 1,3,5,8,12-pentaazacyclotetradecane) containing naphthyl or dansyl subunits can be prepared by template synthesis involving proper sulfonamide derivatives as locking fragments. The macrocyclic complexes are very poorly emissive due to the fluorescence-quenching behavior displayed by Cu^2+^ ions. However, the fluorescence can be recovered as a result of the decomposition of the complexes, which induces the release of free light-emitting subunits to the solution. This reaction takes place very slowly in neutral water but its rate is increased by the presence of sulfite. Therefore, [Cu(azacyclam)]^2+^ derivatives have been investigated as simple chemical probes for the fluorogenic detection of sulfite both on laboratory and real samples. Preliminary tests performed on samples of white wine provided sulfite concentration values that are in agreement with those obtained by a standard analytical method.

## 1. Introduction

Sulfite and related sulfiting agents (i.e., bisulfite and metabisulfite) are well-known as preservatives and antioxidants and are widely used as food and drink additives [1,2,3]. They may sometimes occur spontaneously (i.e., without external addition) in some alimentary products [4,5]. Moreover, sulfite finds application in commercial products other than food and beverages (e.g., cosmetics, drugs), as well as in several industrial processes (e.g., leather, textile, pulp) [3,6]. In addition, sulfur dioxide (SO_2_) is a common air pollutant and can be dissolved in aqueous media with consequent formation of sulfite (SO_3_^2−^) and hydrogensulfite or bisulfite (HSO_3_^−^) at neutral pH. Many studies showed that frequent exposure to SO_2_, as well as to its anionic derivatives, could induce harmful effects on human health, including cardiovascular diseases, neurological disorders, and cancer. Moreover, some individuals exhibit extremely high sensitivity even to very low levels of these compounds [6], resulting in a wide variety of adverse clinical effects that can range from mild-to-life-threatening reactions [3,6,7]. Due to these potential health concerns, in many countries the content of sulfite and/or its related compounds has been strictly limited in recent decades, particularly in foodstuffs and drinks (e.g., wine and beer) [8,9,10,11].

Taking into account the above-mentioned facts, the development of efficient analytical methods for the detection of sulfite has attracted great interest in recent years. Several procedures based on different techniques (e.g., electrochemistry [12], spectrophotometry [13,14], enzyme assay [15,16], chromatography [17,18,19], capillary electrophoresis [20,21], and titration [22,23]) have been reported for the determination of sulfite in various matrices, and specific analytical methods have been developed and extensively used for its monitoring and quantification, particularly in food and wine [23,24,25,26,27,28,29].

Conventional analytical methods often require elaborate sample preparation and suffer from drawbacks such as interference from other sulfur anions, reduced sensitivity at low-sulfite-concentration level, and specificity lacking in some matrices. Moreover, it should be considered that proper experimental conditions must be adopted during the analysis to prevent loss of dissolved sulfite, which can be caused by oxidation (e.g., by O_2_) or by the formation of gaseous SO_2_.

The recent development of chemosensors and chemodosimeters for different species, including anions, offers a novel approach for the detection of a variety of analytes, even in aqueous solution [29,30,31,32,33,34].

Chromofluorogenic detection usually provides some important advantages in terms of sensitivity, selectivity, and simplicity of operation, when compared to classic analytical procedures. Therefore, optical chemical probes based on different principles have been developed and applied to detect a large number of species in different fields, including environmental science, biology and medicine [35,36,37,38].

In recent years, a number of molecular probes for the chromofluorogenic detection of sulfite have been reported. Most of them are based on the nucleophilic behavior of SO_3_^2−^/HSO_3_^−^, in particular towards aldehydes [39,40,41,42], double bonds [43,44,45,46,47,48,49], and levulinate esters [50,51,52,53].

We herein report a couple of simple chemical probes for the selective fluorogenic detection of sulfite in aqueous solution, which are based on copper(II) azacyclam complexes. Azacyclam ligands are 14-membered macrocyclic species [54], whose metal complexes are isostructural with those of cyclam. In fact, four secondary amino nitrogen atoms coordinate the metal center according to a square-planar geometry, while a fifth nitrogen atom does not take part in coordination and can be bound to a variety of functional groups. Similarly to cyclam analogues, azacyclam complexes exhibit both a strong thermodynamic stability and inertness toward ligand dissociation [55,56]. Metal complexes of azacyclam and of other polyaza-macrocyclic ligands have been extensively investigated over the last years, finding application in different areas, including, for instance, metal-organic-framework (MOF) design [57,58,59,60,61], solid-state conducting materials [62,63], electrocatalysis [64,65], and chemosensors and optical probes [66,67].

Azacyclam complexes can be easily synthesized through a template reaction [68] involving the acyclic ligand 2.3.2-tet (1,9-diamino-3,7-diazanonane), a transition metal ion (usually Ni^2+^ or Cu^2+^), formaldehyde, and a “locking fragment” (***LF***), which can be a primary amine [59,60,69,70,71,72,73], an amide [55,74,75,76], or sulfonamide [55,74,77,78], as well as a urea derivative [79]. ***LF***s behave as formal diprotic acids and react by firmly locking the tetraamine chain around the metal ion, thus yielding the macrocyclic structure. In recent years, the template synthesis of azacyclam complexes has been used to prepare a large variety of functionalized macrocyclic compounds and several molecular devices containing metallo-macrocycle subunits [54,55,76,77,78,80].

A limited number of azacyclam complexes bearing light-emitting subunits have been reported so far [55,74,78]. Their emission properties are controlled by the coordinated metal center and its oxidation state [78]. In particular, due to the well-known fluorescence-quenching behavior of copper(II) [81,82,83,84], [Cu(azacyclam)]^2+^ complexes prepared with luminescent ***LF***s display only a very poor emission.

On the other hand, when such [Cu(azacyclam)]^2+^ complexes undergo decomposition (induced by any macrocycle-opening reaction) the release of the light-emitting locking fragments should take place and an intense turn-on of the emission could be expected.

**Scheme 1:**
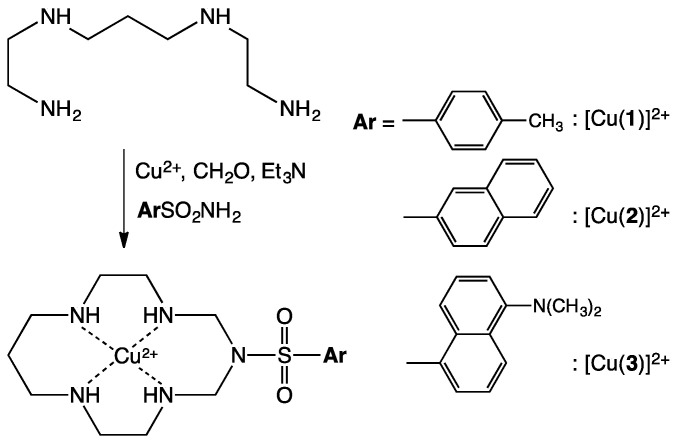
Template synthesis of copper(II) azacyclam complexes.

Starting from this assumption, we investigated the behavior of three different azacyclam complexes, namely [Cu(**1**)]^2+^, [Cu(**2**)]^2+^, and [Cu(**3**)]^2+^, which have been prepared by using *p*-toluenesulfonamide, 2-naphthalenesulfonamide, and dansylamide as ***LF***, respectively (Scheme 1). All the considered compounds undergo decomposition in the presence of sulfite/hydrogensulfite, while other common anionic analytes do not affect their stability. The decomposition of macrocycles **2** and **3** induces the release of fluorescent species and the consequent fluorescence revival can be exploited for the quantitative detection of SO_3_^2−^/HSO_3_^−^ in solution.

## 2. Results and Discussion

### 2.1. Synthesis and Crystal Structure of the Azacyclam Complexes

The copper complexes of azacyclam ligands **1**–**3** were prepared according to the previously reported metal-template route, which is depicted in Scheme 1 [65]. In particular, an EtOH solution of [Cu(2.3.2-tet)]^2+^ complex (prepared in situ; 2,3,2-tet = N,N′-bis(2-aminoethyl)propane-1,3-diamine) was moderately heated and stirred in a common round-bottom flask while the other reagents (locking fragment, base, and formaldehyde) were consecutively added. The macrocyclic copper(II) complexes were isolated as pink-violet solids, usually formed at the end of the reaction (3–7 days) or after addition of excess concentrated perchloric acid to the deep blue-violet reaction mixtures. The addition of HClO_4_ induces the decomposition of possible open-chain by-products and favors the precipitation of the azacyclam complexes as perchlorate or nitrate salts.

When dissolved in water (concentration lower than 10^−3^ M), copper(II) complexes of ligands **1**–**3** display strong absorptions in the UV region (ε = 1500–75000), which are due to the aromatic residues belonging to the locking fragments, and a distinctly less intense band (ε = 76–83 M^−1^cm^−1^) centered between 500 and 550 nm. Solutions with different concentrations (about 10^−5^ and 10^−3^ M) of the complexes were examined in order to accurately detect both the bands, which display very different intensities. The absorption spectra of the investigated azacyclam complexes taken in the 200–400 nm (about 10^−5^ M) as well as in the 400–800 nm range (about 10^−3^ M) are reported in Figures S1–S3. The weak band observed in the visible region results from an envelope of the three possible d–d transitions (xz, yz→x^2^-y^2^, z^2^→x^2^-y^2^, and xy→x^2^-y^2^) in the square-planar copper(II)-tetramine chromophore [85,86]. Spectral data of the investigated complexes as well as of [Cu(2.3.2-tet)]^2+^, taken as a reference, are resumed in Table 1. The macrocyclic complexes display a very similar absorption in the visible range both concerning the maximum wavelength and the intensity, indicating that the square-planar coordination environment is poorly affected by the different structures of the ***LF*** moieties. On the contrary, the d-d band energy of [Cu(**1**)]^2+^, [Cu(**2**)]^2+^, and [Cu(**3**)]^2+^ is distinctly higher than [Cu(2.3.2-tet)]^2+^, due to the macrocyclic nature of azacyclam ligands, which provide a stronger ligand field than the acyclic tetra-amine.

**Figure 1:**
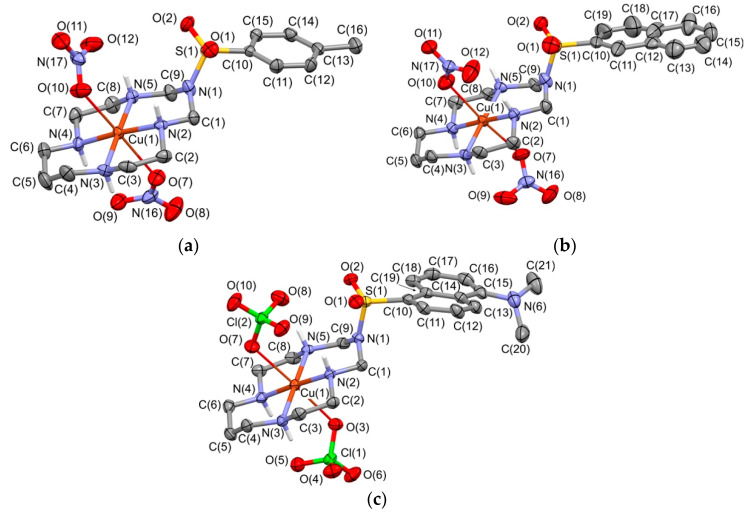
Plot showing thermal ellipsoids for one of the three independent [Cu(**1**)](NO_3_)_2_ azacyclam complexes (**a**), for one of the three independent [Cu(**2**)](NO_3_)_2_ azacyclam complexes (**b**) and for the [Cu(**3**)](ClO_4_)_2_ molecular complex (**c**). Ellipsoids are drawn at the 30% probability level; only H bonded to the secondary amines are shown. The equatorial Cu-N distances are in the range 2.00(1)–2.04(1) Å, whereas the axial Cu-O distances are in the range 2.44(1)–2.62(1) Å; these weak Cu-O bond interactions are drawn as thin sticks.

Crystals containing the [Cu(**1**)]^2+^, [Cu(**2**)]^2+^, and [Cu(**3**)]^2+^ molecular cations suitable for X-ray diffraction study were obtained by slow vapor exchange between ethanol and saturated aqueous solutions of the complexes (filtered immediately before starting the crystallization). In particular, [Cu(**1**)]^2+^ molecular cation occurs as a nitrate salt, whose crystal structure is constituted by three similar but not symmetrically equivalent azacyclam ligands. In addition, the [Cu(**2**)]^2+^ molecular cation forms as a nitrate salt with additional water-solvent molecules and, as [Cu(**1**)]^2+^, the crystal structure contains three independent azacyclam molecular complexes. On the contrary, the [Cu(**3**)]^2+^ molecular cation forms as a perchlorate salt, and its crystal structure is made by a single independent azacyclam molecular complex. Plots showing thermal ellipsoids for one of the three independent [Cu(**1**)](NO_3_)_2_ molecular complexes, for one of the three independent [Cu(**2**)](NO_3_)_2_ molecular complexes, and for the [Cu(**3**)](ClO_4_)_2_ molecular complex are reported in Figure 1 (plots of the remaining independent molecular complexes are reported in Figure S4).

All azacyclam ligands are arranged accordingly to a *trans*-III (RRSS) configuration, with the copper(II) centers bonded to the four secondary amines according to a square-planar coordination geometry. Cu-N bond distances are in the range 2.00(1)–2.04(1) Å for the three independent [Cu(**1**)]^2+^ molecular cations, 2.00(1)–2.03(1) Å for the three independent [Cu(**2**)]^2+^ molecular cations, and 2.01(1)–2.03(1) Å for the [Cu(**3**)]^2+^ molecular cation. These distances are close to the strain-free Cu-N bond in cyclam and cyclam-like macrocycles (2.03 Å) [87].

The tertiary amine of each azacyclam ligands is far from the metal center, with Cu-N_tertiary_ distances in the range 3.21(1)–3.22(1) Å for the [Cu(**1**)]^2+^ molecular cations, 3.22(1)–3.24(1) Å for the [Cu(**2**)]^2+^ molecular cations, and 3.24(1) Å for the [Cu(**3**)]^2+^ molecular cation. Such distances preclude any significant Cu(II)-N_tertiary_ interaction in the azacyclam ligand. However, the observed distances are shorter than the corresponding values of 3.34 Å observed in cyclam-like compounds [88] where a CH_2_ group takes the place of the tertiary amine. Such behavior was already observed and described in the nickel(II) complexes of ligands **1** and **3** [55,78] for which Ni-N_tertiary_ distances are in the range 3.20(1)–3.28(1) Å and it characterizes azacyclam Cu^II^ and Ni^II^ complexes having the tertiary amine connected to electron-withdrawing functional groups.

In all the investigated metal complexes, the Cu^II^ metal center remains in the best plane of the equatorial coordinated amines (deviation from the N_4_ best plane are in the range 0.03(1)–0.07(1) Å) and the copper(II) coordination sphere is completed by two oxygen atoms belonging to the counterions placed in two axial positions. The Cu-O_axial_ distances are longer than the Cu-N_equatorial_ ones, being in the range 2.44(1)–2.57(1) Å for [Cu(**1**)]^2+^, 2.48(1)–2.56(1) Å for [Cu(**2**)]^2+^, and 2.57(1) and 2.62(1) Å for [Cu(**3**)]^2+^ molecular cation. Considering additionally these weak axial bond interactions, the metal centers result in being coordinated six-fold and arranged in an axially elongated octahedral geometry.

The presence of ***LF*** with aromatic moieties favors at the solid state the formations of supramolecular π-stacking interactions. In particular, both the 3[[Cu(**1**)](NO_3_)_2_] and 3[[Cu(**2**)](NO_3_)_2_]·2H_2_O] crystals exhibit face-to-face π-stacking interactions involving the aromatic moieties of the ***LF***: the centroid–centroid separations are in the range 3.58–3.99 Å and the dihedral angles between adjacent aromatic groups are in the range 3.3–11.6°. These π-stacking interactions originate supramolecular chains extending along the direction of the b crystallographic axis (Figure S5). In both these crystals, further intermolecular connections are provided by the nitrate ions, which bridge adjacent molecular compounds by means of N-H···O interactions having the secondary amines of the azacyclam ligands as H-donor species and the O atoms of the nitrate counterions as H-acceptors. These interactions also induce the formation of chains extending along b (Figure S6). The geometrical features of such H-bond interactions are reported in Table S1.

On the contrary, in the [Cu(**3**)](ClO_4_)_2_ crystal the aromatic double ring of the dansyl moieties does not originate face-to-face µ-stacking interactions (the centroid–centroid separation is 4.84 Å and the dihedral angle between adjacent aromatic rings is 40.5°). Moreover, the perchlorate counterions in the [Cu(**3**)](ClO_4_)_2_ crystal are H-bonded only to the secondary amines of the same azacyclam compound and they do not bridge the adjacent molecular compound via perchlorate-mediated supramolecular N-H···O interactions. A simplified view of the H-bonds in the [Cu(**3**)](ClO_4_)_2_ crystal and their geometrical features are reported in Figure S7 and Table S1, respectively.

### 2.2. Decomposition of Azacyclam Complexes in Aqueous Solution

All the investigated compounds are expected to display a strong stability in water, as previously observed for other azacyclam complexes, and more in general, for metal complexes of 14-membered macrocyclic ligands displaying a 5-6-5-6 sequence of chelate rings [86,89,90].

**Figure 2:**
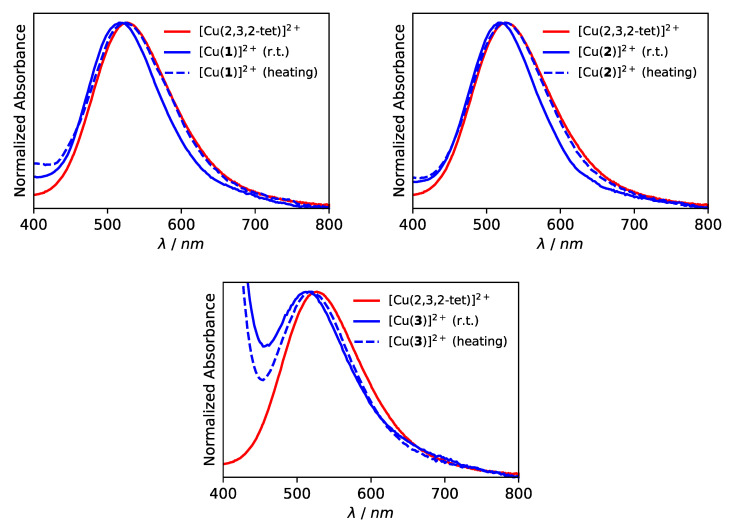
Absorption spectra of complexes [Cu(**1**)]^2+^, [Cu(**2**)]^2+^, and [Cu(**3**)]^2+^ before and after heating compared with the absorption spectrum of [Cu(2,3,2-tet)]^2+^. Absorbances are normalized to facilitate comparison.

The inertness toward demetallation of [Cu(**1**)]^2+^, [Cu(**2**)]^2+^, and [Cu(**3**)]^2+^ is indeed confirmed by the persistence of the d-d band in aqueous solutions within a wide range of pH. For instance, visible spectra measured at room temperature on aqueous solutions of the corresponding perchlorate salts (6–8 × 10^−4^ M) do not undergo significant changes after 24 h at neutral pH (buffered at 7.2) and even in the presence of HClO_4_ (0.1 M). This suggests that, in these conditions, the macrocyclic-complex species do not decompose or decompose to an undetectable extent. Anyway, we performed further investigations in order to check if the stability of the azacyclam complexes in water can be affected by increasing temperature. Therefore, the aqueous solutions of [Cu(**1**)]^2+^, [Cu(**2**)]^2+^, and [Cu(**3**)]^2+^ (6–8 × 10^–4^ M) were buffered at pH 7.2 (to avoid any possible effect of uncontrolled pH), heated for 1 h at 70 °C, and their visible spectra were measured before and after the prolonged heating. This treatment induced in all the samples a small but significant *redshift* (up to 8 nm) of the d-d absorption band (Figure 2). It should be noted that such a batochromic shift may be related to a ligand field weakening in the N_4_ square-planar coordination environment and could be explained on the basis of a replacement of the macrocyclic ligand by an acyclic polyamine species. In fact, the d-d bands in the spectra obtained from the heated solutions of azacyclam complexes match quite well with the visible spectrum of [Cu(2.3.2-tet)]^2+^. This is in agreement with the hypothesis that the macrocyclic compounds decompose, producing the complex with the acyclic tetra-amino ligand. In particular, the d-d band of [Cu(2.3.2-tet)]^2+^ is almost superimposable with those obtained after heating [Cu(**1**)]^2+^ and [Cu(**2**)]^2+^. The lower redshift (about 4 nm) observed in the case of [Cu(**3**)]^2+^ could be tentatively explained considering that the tail of the strong dansyl moiety absorption (λ = 334 nm, ε = 4000 M^−1^cm^−1^) may affect the weak d-d band, which consequently may appear slightly distorted toward lower wavelengths.

Moreover, the absorption spectrum of a [Cu(**3**)]^2+^ aqueous solution (2 × 10^–5^ M) registered after heating (1 h, 70 °C, pH 7.2) in the 200–400 nm range clearly shows a variation of the UV absorption bands when compared with the spectrum before decomposition (see Figure S8). In particular, the spectrum of the decomposed complex is superimposable to that of dansyamide, supporting the hypothesis of the macrocyclic ring opening with consequent release of the LF subunit.

ESI-MS experiments were performed to investigate the identity of the supposed decomposition products. At room temperature, the characteristic peaks ascribed to the macrocyclic complexes (e.g., M^2+^, {M-H}^+^, and {M + anion}^+^ signals) can be observed for [Cu(**1**)]^2+^, [Cu(**2**)]^2+^, and [Cu(**3**)]^2+^ (Figure S9). After heating, these peaks disappear in the MS spectra of the azacyclam complexes and new signals ascribable to the [Cu(2.3.2-tet)]^2+^ complex species can be observed (Figure S10). In particular, the following peaks due to the acyclic complex have been detected: *m*/*z* = 222 ({M-H}^+^ all the spectra), 285 ({M + NO_3_}^+^ in [Cu(**1**)]^2+^ and [Cu(**2**)]^2+^), and 322 ({M + ClO_4_}^+^ in [Cu(**3**)]^2+^, M = [Cu(2.3.2-tet)]^2+^). In addition, signals corresponding to the free-locking fragments can be observed at *m*/*z* = 194, 230, and 273 ({M + Na}^+^, M = *p*-toluensulfonamide, 2-naphthaleneamide, and dansylamide, respectively). In the case of [Cu(**3**)]^2+^, the MS spectrum performed after decomposition also shows peaks at *m*/*z* = 251 ({M + H}^+^) and 501 ({2M + H}^+^, M = dansylamide).

**Figure 3:**
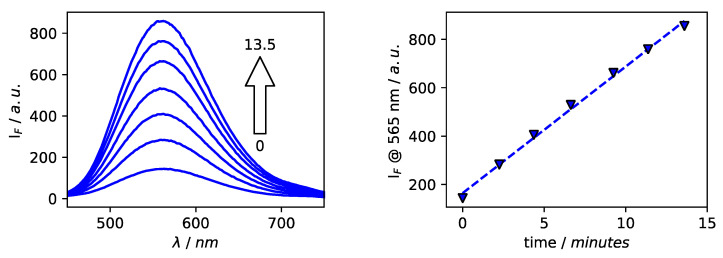
Emission spectra (λ_exc_ = 325 nm) of dansylamide (intensity increasing with time from 0 to 13.5 min) formed during the decomposition of [Cu(**3**)]^2+^ 2 × 10^–5^ M at 70 °C and pH 7.2 (**left**); time-dependent profile of the fluorescence intensity (**right**).

The decomposition of [Cu(**2**)]^2+^ and [Cu(**3**)]^2+^ was also investigated by performing light-emission measurements. As already discussed, these two complexes are very poorly emissive due to the fluorescence quenching induced by copper(II), but after heating, their aqueous solutions (2 × 10^−5^ M) display very intense fluorescence bands at 345 and 565 nm, which correspond to the emission of the locking fragments released in solution, i.e., 2-naphthalenesulfonamide and dansylamide, respectively (Figure S11). The decomposition of [Cu(**3**)]^2+^ has been also monitored by collecting the emission spectra at different times. As an example, a family of dansylamide spectra collected during a monitoring experiment (about 15 min) and the corresponding time-dependent profile of fluorescence intensity, I_F_, are reported in Figure 3.

The above-described experiments clearly indicate that copper(II) azacyclam complexes, which seem to be extremely stable at room temperature, can undergo decomposition at neutral pH by heating, providing the starting compounds used in their template synthesis, i.e., [Cu(2.3.2-tet)]^2+^ and the locking fragments.

**Figure 4:**
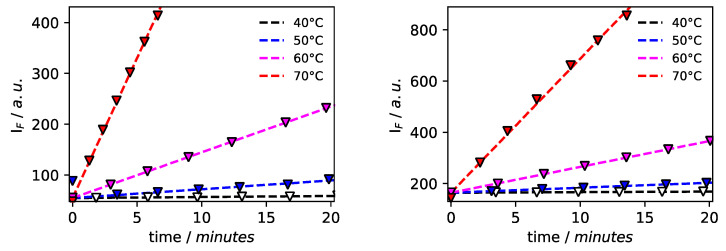
Time-dependent profiles of fluorescence intensity obtained during the decomposition at different temperatures (pH buffered at 7.2) of [Cu(**2**)]^2+^ 2 × 10^–5^ M (**left**) and [Cu(**3**)]^2+^ 2 × 10^–5^ M (**right**).

Other similar experiments have been performed to better assess the effect of temperature on the decomposition process of the azacyclam complexes. In particular, aqueous solutions of [Cu(**2**)]^2+^ and [Cu(**3**)]^2+^, buffered at pH 7.2, were thermostated at temperatures between 40 and 70 °C, while their emission intensity was monitored. All the spectra were collected by using the same instrumental conditions, in order to correctly compare the data obtained in the different monitoring experiments. The I_F_ vs. time profiles obtained at different temperatures are reported in Figure 4. As the time required for the complete decomposition varies a lot depending on the temperature, the time-dependent profiles up to 20 min are reported.

The I_F_ vs. time plots reported in Figure 4 corresponds to the initial straight line of the complete time-dependent I_F_ profiles; therefore, their slopes, dI_F_/dt, are directly related to the rate of the slowest step. As expected, the rate of the decomposition process exhibits an exponential increase with temperature (Figure S12).

**Scheme 2:**
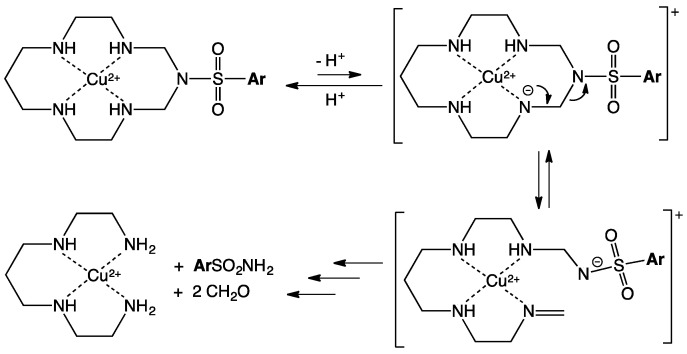
Hypothesized mechanism for the decomposition of [Cu(azacyclam)]^2+^ complexes in water.

On the basis of the above-mentioned results, a plausible mechanism for the decomposition reaction (Scheme 2) can be hypothesized. The first step may consist of the deprotonation of an amine adjacent to the sulfonamide group, also favored by the double-positive charge of the coordinated metal ion. The deprotonated species can then undergo a ring opening step, forming an imine group on an extremity of the chain and the deprotonated sulfonamide group on the other. Afterwards, the decomposition reaction may proceed following the opposite direction of the template synthesis pathway [74], providing again [Cu(2.3.2-tet)]^2+^, formaldehyde, and the original ***LF***s, as demonstrated by MS and emission spectroscopy measurements.

The same final result can be expected considering, as an alternative decomposition route, the direct nucleophilic attack of OH^−^ to the diazamethylene group, as depicted in Scheme S1.

Experiments performed at different pH values (ranging between 6 and 8) and at 40 °C showed that the decomposition rate evaluated by dI_F_/dt, as described above, distinctly increases with pH (Figure S13), supporting both the hypothesized mechanisms of decomposition, which are strongly favored by increasing OH^−^ concentration anyway.

### 2.3. Decomposition in the Presence of Sulfite

**Figure 5:**
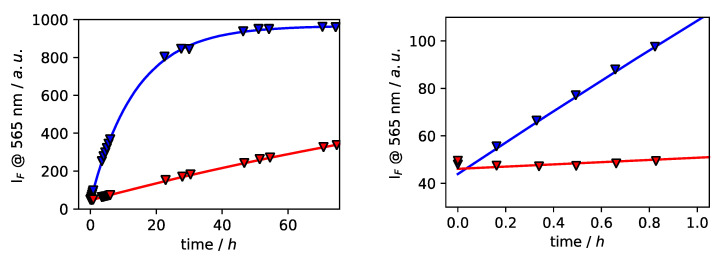
Time-dependent profiles of fluorescence intensity obtained during the decomposition of [Cu(**3**)]^2+^ (2 × 10^−5^ M, pH = 7.2, T = 40 °C) in the presence (blue triangles) and in the absence of sulfite (red triangles). (**Left**) complete profiles (72 h); (**right**) profiles after 1 h monitoring. Blue and red lines represent the fitting results in the presence and in the absence of sulfite, respectively.

The following experiment was preliminarily carried out in order to check the possible effect of sulfite on the decomposition of azacyclam complexes. As the reaction is affected by pH and T, the experiment was performed by carefully controlling the experimental conditions (pH = 7.2, phosphate buffer; T = 40 °C, thermostatic bath). An aqueous solution of [Cu(**3**)]^2+^ (2 × 10^−5^ M) was treated with excess Na_2_SO_3_ (5 × 10^−4^ M) and its emission was monitored over time. It should be noted that at neutral pH, both SO_3_^−^ and HSO_3_^−^ are present in relevant concentrations. For simplicity, the term “sulfite” will be used in the following, bearing in mind the two species in equilibrium. For comparison, a similar experiment was simultaneously performed on another portion of the same complex solution without adding sulfite. The corresponding profiles I_F_ vs. time obtained from the fluorescence monitoring are reported in Figure 5. In the absence of sulfite, dansylamide emission linearly increased with time during the experiment (up to 72 h), although the fluorescence enhancement was almost undetectable after the first hour, coherently with the slow decomposition of complex [Cu(**3**)]^2+^ at 40 °C and neutral pH. On the contrary, in the presence of excess sulfite, the I_F_ enhancement was distinctly larger even at short time, suggesting that the decomposition rate had considerably increased (see inset in Figure 5).

The effect of other anionic species on the decomposition of copper(II) complexes was also investigated.

**Figure 6:**
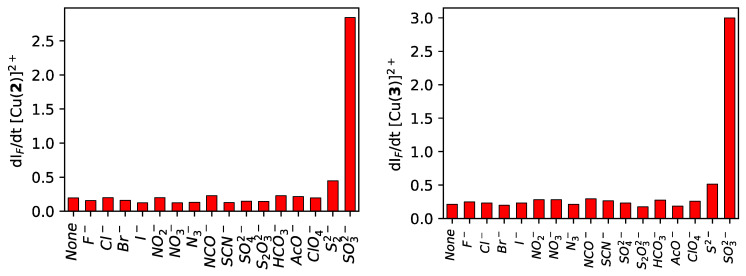
Effect of different anions on the decomposition reaction of [Cu(**2**)]^2+^ (**left**) and [Cu(**3**)]^2+^ (**right**) both 2 × 10^−5^ M (pH = 7.2; T = 40 °C). Anion concentrations were 5 × 10^−4^ M except for sulfide and sulfite (both 2 × 10^−5^ M).

Different portions of [Cu(**2**)]^2+^ and [Cu(**3**)]^2+^ aqueous solutions (2 × 10^−5^ M, pH = 7.2, T = 40 °C) were treated with a variety of anions (fluoride, chloride, bromide, iodide, nitrite, nitrate, azide, cyanate, thiocyanate, sulfate, thiosulfate, sulfide, hydrogencarbonate, acetate, perchlorate) and their fluorescence was monitored. In all the considered solutions the emission intensity increased very slowly and the corresponding time-dependent profiles were very similar to that obtained in the absence of any anion (Figure S14). On the contrary, as mentioned above, the presence of sulfite induced a distinctly higher fluorescence increase even after a short time (e.g., 20 min), suggesting that it is the only anionic species that considerably affects the decomposition of azacyclam complexes. The unique behavior of sulfite can also be clearly inferred from the comparison of the decomposition rates, i.e., dI_F_/dt values obtained from the slope of time-dependent fluorescence profiles, as described in the previous section. A graphical representation of dI_F_/dt values corresponding to the decomposition of [Cu(**2**)]^2+^ and [Cu(**3**)]^2+^ in the presence of the envisaged anions is reported in Figure 6. When sulfite is added to the complex solution, the decomposition rate underwent a ten-fold increase, while most of the other anions did not substantially modify the reaction rate of the complexes at neutral pH.

A somewhat different behavior was observed in the case of HS^−^: the demetalation of macrocyclic Cu^2+^ complexes induced by HS^−^ was previously reported and even exploited for the detection of this anion [82,83,84]; therefore, its effect on the decomposition of [Cu(**2**)]^2+^ and [Cu(**3**)]^2+^ could be expected. To limit the possible interference of HS^−^, its concentration level was kept the same as sulfite in the corresponding competition test. In this situation the effect of HS^−^ is however minimal when compared to HSO_3_^−^/SO_3_^2−^.

**Scheme 3:**
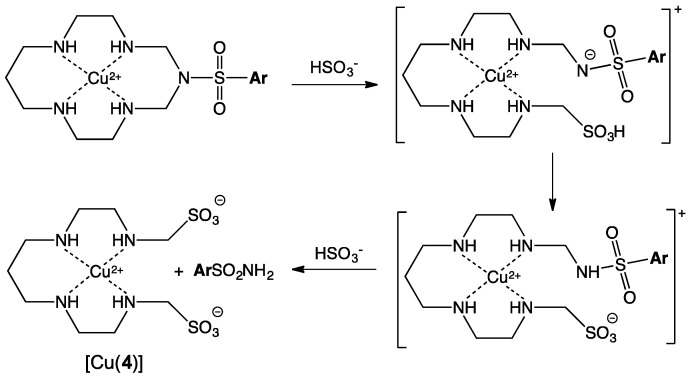
Proposed mechanism of the sulfite-induced decomposition of copper(II) azacyclam complexes. The possible coordination of sulfonate groups in ligand **4** to the copper center is not represented in the scheme.

On the basis of the experimental data, two different mechanisms could be hypothesized in order to explain the role of sulfite in the decomposition reaction of macrocyclic complexes:

- coordination of HSO_3_^−^/SO_3_^2−^ to Cu^2+^, which could favor the reduction of the coordinated metal center and the formation of poorly stable copper(I) intermediates. The macrocyclic [Cu(N_4_)]^+^ species easily undergoes demetalation, as the square-planar coordination environment does not sufficiently stabilize that oxidation state. As a result of this mechanism, the unstable free ligand can be hydrolyzed generating the free sulfonamide ***LF***. Although it could seem plausible, this hypothesis is not completely convincing because sulfite is not effective in the decomposition of macrocyclic copper(II) complexes other than azacyclam ones (e.g., derivatives of [Cu(cyclam)]^2+^) [83];
- nucleophilic reaction of sulfite and methylenediamine groups of the azacyclam framework, with consequent formation of aminomethanesulfonate derivatives, and release of sulfonamide locking fragments (Scheme 3).

In order to check the second hypothesis of the mechanism, the sulfite-induced decomposition was studied in more detail. In particular, a buffered (pH = 7.2) aqueous solution of [Cu(**1**)]^2+^ (2.6 × 10^−3^ M) was treated with excess Na_2_SO_3_ (2.6 × 10^−2^ M) at 40 °C and the resulting solution was analyzed after 2 h by mass spectroscopy. The ESI-MS spectrum exhibits a peak at *m*/*z* 194, corresponding to {Toluenesulfonamide + Na}^+^ and a base peak at *m*/*z* 432, which corresponds to the {[Cu(**4**)]^0^ + Na}^+^ species, i.e., to the neutral bis-aminomethanesulfonate acyclic complex hypothesized as decomposition product in Scheme 3. The same peak at *m*/*z* 432 can be also observed in the ESI-MS spectrum obtained after carrying out the same experiment on [Cu(**2**)]^2+^ and [Cu(**3**)]^2+^ (2.6 × 10^−3^ M), in addition to the peaks related to the free fluorophores (2-Naphthalenesulfonamide and dansylamide, respectively), supporting again the mechanism proposed in Scheme 3. ESI-MS spectra obtained after decomposition of [Cu(**1**)]^2+^, [Cu(**2**)]^2+^, and [Cu(**3**)]^2+^ in the presence of excess sulfite are reported in Figure S15.

Moreover, after treatment with sulfite, the absorption spectrum of the resulting solution in the visible range showed a broad band centered at 568 nm (ε = 117 M^−1^cm^−1^, Figure S16). Notably, the redshift of the d-d band is considerably higher than that observed after the decomposition in neutral water or in the presence of OH^−^, suggesting again that a copper complex different from [Cu(2,3,2-tet)]^2+^ is obtained after decomposition induced by HSO_3_^−^/SO_3_^2−^. The bis-aminomethanesulfonate acyclic ligand **4**, i.e., the hypothesized final product of sulfite-induced azacyclam decomposition (Scheme 3), should provide a weaker ligand field than 2,3,2-tet due to the electron-withdrawing character of sulfonate, thus it could induce a larger shift of the d-d band towards lower energy. Moreover, we cannot completely rule out the formation of different coordination species (i.e., penta-coordinated or distorted octahedral hexa-coordinated) in which oxygen atoms of sulfonate groups are involved in the coordination to copper(II). The presence of different coordination species may result in the overlapping of several d-d absorptions, with consequent broadening and significant redshift of the resulting band. The coordination of aminomethylsulfonate group to copper(II) ion according to the chelate mode in hexacoordinated complexes has been already reported in literature [91].

### 2.4. Quantitative Detection of Sulfite

**Figure 7:**
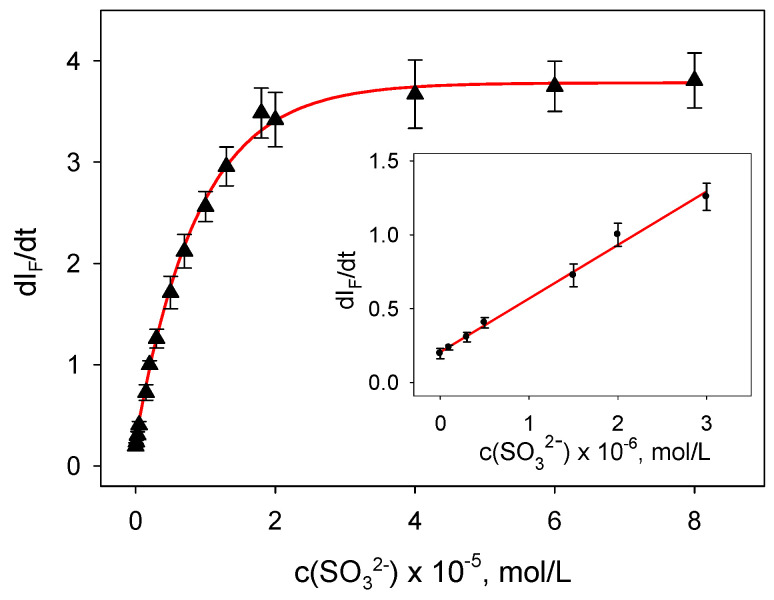
Effect of sulfite concentration on the decomposition rate of [Cu(**2**)]^2+^ (black triangles: experimental data; red line: fitting curve). Profile at lower concentration values is reported in the inset.

The experiments described above demonstrated that sulfite significantly increases the decomposition rate of azacyclam complexes. When considering complexes [Cu(**2**)]^2+^ and [Cu(**3**)]^2+^, the fluorescence enhancement due to the release of the fluorophore can be in principle exploited to signal the presence of sulfite in solution. However, the decomposition of azacyclam complexes, even in the presence of sulfite, is a quite slow process (see Figure 5) and the related variation of I_F_ at short times (i.e., <1 h) cannot be reliably used for analytical application. On the other hand, the decomposition rate, dI_F_/dt, is strongly affected by sulfite and could be usefully related to the concentration of this species in solution. To demonstrate the relationship between dI_F_/dt and [SO_3_^2−^], the decomposition of [Cu(**2**)]^2+^ was investigated in the presence of an increasing amount of sodium sulfite under the same experimental conditions described above (pH = 7.2; T = 40 °C). The fluorescence intensity at 345 nm (λ_exc_ = 275) was monitored at the initial stage of the reaction (20 min) in each experiment, and the dI_F_/dt value corresponding to each considered [Na_2_SO_3_] value was determined from I_F_ vs. time profile. The resulting graph obtained by plotting dI_F_/dt vs. sulfite concentration is reported in Figure 7. The decomposition rate increases with sulfite concentration until this is almost equivalent to the concentration of azacyclam complex; then, it reaches a “plateau” and does not undergo further significant changes.

**Figure 8:**
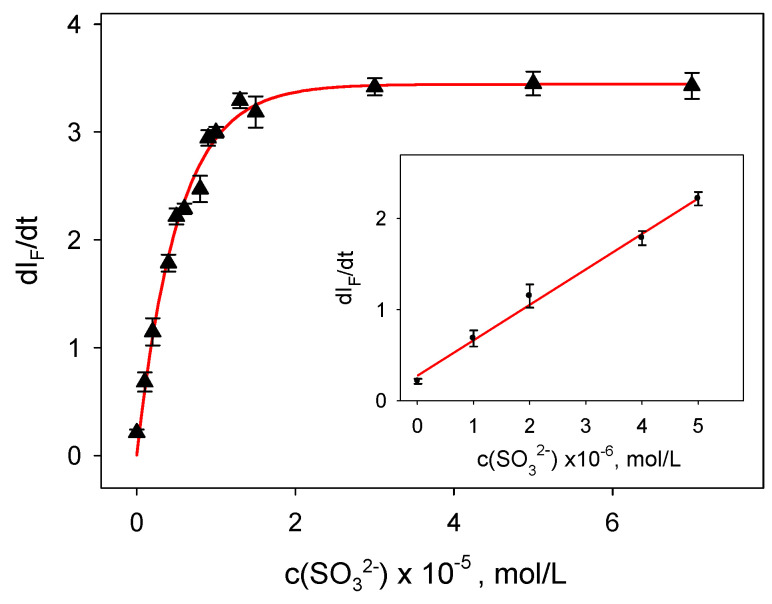
Effect of sulfite concentration on the decomposition rate of [Cu(**3**)]^2+^ (black triangle—experimental data; red line—fitting curve). Profile at lower concentration values is reported in the inset.

A satisfactory fit of the experimental data was obtained by using Equation (1):

(1) $$\left(\frac{dI_{F}}{dt}\right)=v_{0}+a\cdot \left(1-e^{-b\cdot c\left({\mathrm{SO}}_{3}^{2-}\right)}\right)$$

where *v*_0_ is the reaction rate in the absence of SO_3_^2−^, *a* is the difference between the “plateau rate”, and *v*_0_, *b* is a parameter used to describe the curvature and *c*(SO_3_^2−^) is the global concentration of sulfites ([HSO_3_^−^] + [SO_3_^2−^], expressed in mol/L in the following data). The resulting curve matches the experimental points in the investigated concentration range. The same behavior was also observed for [Cu(**3**)]^2+^ (Figure 8). The fitting data for both compounds are resumed in Table S2.

Although the overall response of both probes is nonlinear, the dI_F_/dt vs. *c*(SO_3_^2–^) profiles are almost linear at lower concentration values (see insets in Figure 7 and Figure 8) and can be properly used as calibration lines for the quantitative determination of sulfite roughly in the 0.5–10 μM range. Therefore, possible sensitivity problems when analyzing samples with higher sulfite concentration can be easily circumvented by diluting the sample solution (e.g., with a pH buffer) before starting the analysis.

Limit of detection (LOD) and limit of quantification (LOQ) values were also estimated for both probes [92] and are reported in Table S2. Interestingly, a detection limit as low as 30 ppb (calculated as SO_2_) was found.

**Figure 9:**
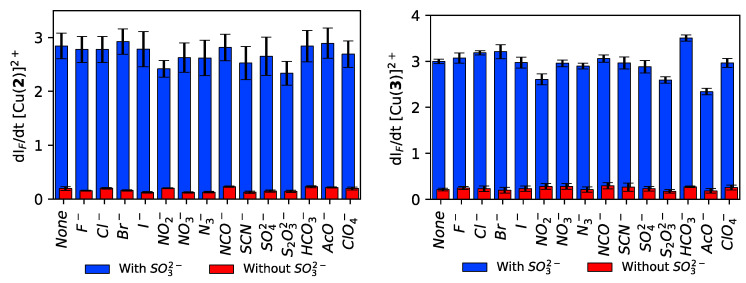
Effect of different anions on the decomposition rate of [Cu(**2**)]^2+^ (**left**) and [Cu(**3**)]^2+^ (**right**) in the presence of 1 × 10^−5^ M SO_3_^2−^ (blue bars) compared to the above-reported data in the absence of SO_3_^2−^ (red bars).

In order to check any possible interference of other anions on the sulfite-induced decomposition rate, more experiments with a fixed concentration of SO_3_^2−^ (1 × 10^−5^ M) and the simultaneous presence of a second anion (5 × 10^−4^ M) were performed. In general, the dI_F_/dt data obtained by these tests were very similar to the decomposition rate in the presence of sulfite alone (Figure 9). For [Cu(**2**)]^2+^ a slight decrease was observed in the case of S_2_O_3_^2−^ and NO_2_^−^, while in the case of [Cu(**3**)]^2+^ acetate (decrease) and hydrogencarbonate (increase) also induced a small but detectable change with respect to the sulfite alone.

Further experiments were performed in order to test the applicability of the above-described method to evaluate the concentration of sulfite in real samples.

Sulfite is generally present in three forms in food or beverage systems: free, reversibly bound, and irreversibly bound. Free sulfite, as its name suggests, is not bound to any other component and can be more easily quantified. Reversibly and irreversibly bound sulfites exist when adducts form between sulfite and various food or beverage components (i.e., acetaldehyde, sugar monomers, or sugar acids) [2].

Determination of bound sulfite is usually not straightforward because their quantification requires particular pretreatment of samples or is scarcely reliable in the presence of irreversibly bound sulfite derivatives, which are usually very stable. Therefore, the check of the present method was limited to the determination of free sulfite in a sample of a popular branded white wine.

At first, the content of sulfite in the selected wine was determined by the widely used Ripper standard method [23,25], and a concentration of 39 ± 5 mg/L (calculated as SO_2_, corresponding to about 4.9 × 10^−4^ M) was found. Then, different samples of wine were properly diluted in order to obtain a sulfite concentration in the range corresponding to the “linear” portion of profiles described by Equation (1) (final dilution ratio 1:100) and treated with [Cu(**2**)]^2+^ or [Cu(**3**)]^2+^ (so as to obtain a final concentration of metal complex of about 2 × 10^−5^ M). Determination of free sulfite was performed according to the above-described experimental procedure (pH = 7.2, T = 40 °C). In particular, fluorescence intensity at 345 (for [Cu(**2**)]^2+^) or at 565 nm (for [Cu(**3**)]^2+^) was monitored for about 20 min and the corresponding dI_F_/dt value was used to obtain sulfite concentration. Typical spectra families and the corresponding I_F/_ vs. *t* plots obtained from the experiments performed with naphthyl and dansyl probes are reported in Figures S17 and S18, respectively. Sulfite-concentration values determined by applying this fluorogenic method on the diluted wine samples were 6.5 ± 0.5 × 10^−6^ and 6.4 ± 0.5 × 10^−6^ M, by using [Cu(**2**)]^2+^ or [Cu(**3**)]^2+^, respectively. These concentration values correspond to 41 ± 4 and 41 ± 2 mg/L (calculated as SO_2_), respectively, in the original wine samples, and are in good accordance with the value obtained by the standard method.

## 3. Materials and Methods

### 3.1. Materials and General Procedures

Unless otherwise stated, all reagents and solvents were supplied by Sigma-Aldrich and used as received.

Concentrated solutions of the complexes were stored in a refrigerator to ensure long-term stability.

UV-vis absorption spectra were recorded on a Cary 50 (Varian Ltd., Mulgrave, Victoria, Australia) spectrophotometer using a 1 cm path-length optical-glass cuvette. Emission spectra were recorded on a Cary Eclipse (Varian Ltd., Mulgrave, Victoria, Australia) spectrofluorimeter using a 1 cm × 1 cm quartz cuvette; the spectra were smoothed using a 5 points Savitzky–Golay filter [93]. ESI-MS spectra were recorded on a Thermo-Finnigan LCQ Advantage Max (Thermo Electron, San Josè, CA, USA) equipped with an ESI source. IR spectra were recorded on a Spectrum 100 FT-IR (PerkinElmer, Waltham, MA, USA) spectrometer equipped with an ATR accessory.

Computations and data plotting were performed using the SciPy ecosystem [94,95,96,97]. Nonlinear regressions were performed using the Lmfit library [98].

### 3.2. Synthesis

[Cu(**1**)](NO_3_)_2_ and [Cu(**2**)](NO_3_)_2_ were prepared following the previously reported procedure [74].

Synthesis of [Cu(**3**)](ClO_4_)_2_ (3-dansyl-1,3,5,8,12-pentaazacyclotetradecane copper(II) perchlorate) was performed following a similar procedure. Cu(NO_3_)_2_·3H_2_O (1.0 mmol) was dissolved in EtOH (6 mL) in a round-bottom flask and 2,3,2-tet (1.0 mmol diluted in a small amount of EtOH) was added dropwise while stirring. Dansylamide (1.0 mmol) suspended in a EtOH:MeCN mixture was added, followed by Et_3_N (0.15 mL) and 37% aqueous CH_2_O (0.4 mL). The resulting mixture was kept at 50–60 °C for 7 days with continuous stirring. Further, 37% aqueous CH_2_O (0.2 mL) was added every day for the first 4 days. After a week, the purple solution was slightly concentrated with a rotary evaporator and cooled in an ice bath. 30% HClO_4_ was slowly added while stirring until an abundant precipitate was obtained. The solid was collected using a Buchner funnel, washed with several portions of cold EtOH followed by cold diluted HClO_4_ and finally with another portion of cold EtOH. The pink powder was further suspended in THF, filtered, and washed with the same solvent in order to ensure the complete removal of any unreacted dansylamide. Finally, the product was dried under vacuum (yield 30%). ESI-MS (MeOH): *m*/*z* = 496.2 ([M-H]^+^, 100%). FT-IR (ATR, cm^−1^): 3229 (N-H); 1335, 1304, 1148 (S = O); 1084 (ClO_4_^−^); 936 (S-N). Elem. Anal.: calcd (%) for C_21_H_34_Cl_2_CuN_6_O_10_S: C 36.19, H 4.92, N 12.06; found C 36.13, H 4.95, N 12.01.

### 3.3. X-ray Crystallographic Study

Crystals of the complex salt 3[[Cu(**1**)](NO_3_)_2_], 3[[Cu(**2**)](NO_3_)_2_]·2H_2_O] and [Cu(**3**)](ClO_4_)_2_, suitable for crystallographic studies, were obtained by slow diffusion of ethanol vapors into aqueous solutions of the complexes.

Diffraction data for the 3[[Cu(**1**)](NO_3_)_2_] crystal (reddish-violet, prismatic, 0.60 × 0.60 × 0.51 mm^3^) were collected by means of a Enraf-Nonius CAD4 four-circle diffractometer (Enraf-Nonius, Delft, The Netherlands) equipped with a punctual detector (scintillation counter), whereas diffraction data for both 3[[Cu(**2**)](NO_3_)_2_]·2H_2_O] (reddish-violet, prismatic, 0.45 × 0.18 × 0.13 mm^3^) and [Cu(**3**)](ClO_4_)_2_ crystals (pale orange, platy prismatic, 0.50 × 0.20 × 0.08 mm^3^) were collected on a Bruker AXS CCD-based three-circle diffractometer (Bruker AXS Inc., Madison, Wisconsin, USA). Both instruments work at ambient temperature with graphite-monochromatized Mo K*α* X-radiation (λ = 0.7107 Å). Crystal data for the studied molecular compounds are reported in Table 2.

Data reduction (including intensity integration, background, Lorentz, and polarization corrections) for intensities collected with the conventional diffractometer was performed with the WinGX package [99]. Absorption effects were evaluated with the psi-scan method [100] and absorption correction was applied to the data. Frames collected by the CCD-based system were processed for data reduction with the SAINT software [101] and intensities were corrected for Lorentz and polarization effects. Absorption effects were empirically evaluated by SADABS software [102] and absorption corrections were applied to the data. All crystal structures were solved by direct methods (SIR 97) [103] and refined by full-matrix least-squares procedures on *F*^2^ using all reflections (SHELXL 2018/3) [104]. Positions for hydrogens bonded to the secondary amines were identified in the final ∆*F* maps and refined, restraining the N-H distances to be 0.90 ± 0.01 Å Other hydrogen atoms were placed at calculated positions with the appropriate AFIX instructions and were refined using a riding model. Anisotropic displacement parameters were refined for all non-hydrogen atoms. For the 3[[Cu(**2**)](NO_3_)_2_]·2H_2_O] crystal, soft restraints on the molecular geometry (SAME) and enhanced rigid bond restraints (RIGU) were applied for a naphthalene aromatic ring, probably affected by unresolved positional disorder. Positions for protons belonging to water solvent molecules remained undetermined. CCDC 2150891, 2150892, and 2150893 contain the supplementary crystallographic data for this paper. These data can be obtained free of charge from the Cambridge Crystallographic Data Centre.

## 4. Conclusions

Macrocyclic copper(II) complexes, i.e., [Cu(azacyclam)]^2+^, can be easily prepared by a template reaction involving a variety of locking fragments, including fluorescent ones. Complexes [Cu(**2**)]^2+^ or [Cu(**3**)]^2+^, although functionalized with naphthyl and dansyl moiety, respectively, do not display light-emitting properties due to the fluorescence quenching process activated by the copper(II) ions. Nevertheless, the typical emission of 2-naphthalenesulfonamide and dansylamide used as locking fragments in the template synthesis of the macrocyclic compounds can be recovered as a result of complex decomposition. In fact, the ring-opening process takes place with the simultaneous release of the locking fragments and the consequent turn-on of their fluorescence. The decomposition is extremely slow in neutral water at room temperature but its rate significantly increases at basic pH and/or at high temperature, as demonstrated by different spectroscopic investigations. Interestingly, decomposition of the macrocyclic species was observed to be strongly increased in the presence of sulfite at neutral pH. The reaction with SO_3_^2−^ was apparently not observed before in analogous Cu^2+^ macrocyclic complexes (e.g., [Cu(cyclam)]^2+^ derivatives) and may be related to the unique structure of azacyclam compounds. No other anion affected the complexes’ decomposition, apart from sulfide, which induced a moderate increase in the reaction rate, albeit distinctly lower than sulfite. Therefore, the copper(II) azacyclam complexes were investigated as possible chemical probes displaying a fluorescence-turn-on behavior towards sulfite. In particular, in order to gain a faster response, the correlation between decomposition rate (dI_F_/dt) and sulfite concentration was exploited to perform quantitative determination of this anionic substrate on laboratory as well as real samples. Although the nonlinear response of these systems can induce a decrease in sensitivity with increasing SO_3_^2−^ concentrations (ultimately leading to an upper concentration limit), in practice this limit may be easily circumvented by diluting the sample solution (e.g., with a pH buffer).

Preliminary tests on white-wine samples provided free sulfite-concentration values that were confirmed by independent analyses performed by the standard Ripper method.

In conclusion, this work has shown that [Cu(azacyclam)]^2+^ complexes, which are extremely inert toward decomposition in very acidic solution, can decompose in the presence of sulfite and this reaction can be usefully exploited to develop a fluorogenic procedure for the selective detecting of SO_3_^2−^. This procedure is based on a novel approach and may represent an interesting addition to the existing sulfite-determination methods.

## Figures and Tables

**Table 1:** UV-vis spectral data for copper(II) azacyclam complexes and reference complex with acyclic ligand 2.3.2-tet.

Compound	λ (nm)	ε (M^−1^cm^−1^)
[Cu(**1**)]^2+^	231 265(sh) 518	19,500 9000 79
[Cu(**2**)]^2+^	232 265 327 519	75,000 12,000 1500 83
[Cu(**3**)]^2+^	250 334 517	23,000 4000 76
[Cu(2.3.2-tet)]^2+^	526	73

**Table 2:** Crystal data for investigated crystals.

	3[[Cu(1)](NO_3_)_2_]	3[[Cu(2)](NO_3_)_2_]·2H_2_O]	[Cu(3)](ClO_4_)_2_
Formula	C_48_H_87_Cu_3_N_21_O_24_S_3_	C_57_H_91_Cu_3_N_21_O_26_S_3_	C_21_H_34_Cl_2_CuN_6_O_10_S
*M*	1629.21	1773.31	697.05
Crystal system	orthorhombic	orthorhombic	monoclinic
Space group	*Pbca* (no. 61)	*Pbca* (no. 61)	*P* 2_1_/*c* (no. 14)
*a* [Å]	22.803(3)	24.4069(11)	13.4017(9)
*b* [Å]	21.832(6)	21.8999(10)	26.1598(18)
*c* [Å]	27.537(5)	28.2847(13)	8.2937(6)
*β* [°]	90	90	101.2578(11)
*V* [Å³]	13709(5)	15118.4(12)	2851.7(3)
*Z*	8	8	4
*ρ*_calcd_ [g cm^−3^]	1.579	1.558	1.624
*µ* Mo_K__α_ [mm^−1^]	1.105	1.011	1.090
Scan type	ω scans	ω scans	ω scans
θ range [°]	2.0–25.1	1.4–25.1	1.7–25.0
Measured reflections	12185	132295	26809
Unique reflections	12185	13361	5016
*R_int_*	-	0.032	0.025
Strong data [*I*_O_ > 2σ(*I*_O_)]	10116	10033	4364
Refined parameters	928	1027	384
*R1, wR2* (strong data)	0.0598, 0.1177	0.0613, 0.1579	0.0324, 0.0859
*R1, wR2* (all data)	0.0724, 0.1262	0.0839, 0.1765	0.0380, 0.0904
GOF	1.134	1.042	1.061
Max/min residuals [eÅ^−3^]	0.48/−0.37	1.76/−0.66	0.48/−0.32

## Data Availability

The data used to support the findings of this study are included within the article and in Supplementary Materials.

## Supplementary Materials

The following supporting information can be downloaded at: https://www.mdpi.com/article/10.3390/molecules27061852/s1, Figure S1: Absorption spectra of complex [Cu(**1**)]^2+^ in aqueous solution; Figure S2: Absorption spectra of complex [Cu(**2**)]^2+^ in aqueous solution; Figure S3: Absorption spectra of complex [Cu(**3**)]^2+^ in aqueous solution; Figure S4: Plot showing thermal ellipsoids for the other independent azacyclam ligands occurring in the 3[[Cu(**1**)](NO_3_)_2_] and 3[[Cu(**2**)](NO_3_)_2_]·2H_2_O]; Figure S5: Simplified sketches of the supramolecular chains maintained by face-to-face p-stacking interactions in the 3[[Cu(**1**)](NO_3_)_2_] and 3[[Cu(**2**)](NO_3_)_2_]·2H2O] crystals; Figure S6: Simplified sketches of the N-H···O H-bonds in the 3[[Cu(**1**)](NO_3_)_2_] and 3[[Cu(**2**)](NO_3_)_2_]·2H_2_O] crystals; Figure S7: A simplified sketch of the N-H···O H-bonds occurring in the [Cu(**3**)](ClO_4_)_2_ crystal; Table S1: Geometrical features of the N-H···O H-bonds occurring in the studied crystals; Figure S8: Absorption spectra of complex [Cu(**3**)]^2+^ before and after decomposition; Figure S9: ESI-MS spectra of aqueous solutions of [Cu(1)](NO_3_)_2_, [Cu(**2**)](NO_3_)_2_, and [Cu(3)](ClO_4_)_2_; Figure S10: ESI-MS spectra of aqueous solutions of the investigated copper(II) azacyclam complexes after 1 h heating at 70 °C; Figure S11: Emission spectra of aqueous solutions of [Cu(**2**)]^2+^ and [Cu(**3**)]^2+^ taken at room temperature and after 1 h heating at 70 °C; Figure S12: Effect of temperature on the decomposition rate of [Cu(**2**)]^2+^ and [Cu(**3**)]^2+^ in water at pH 7.2; Figure S13: Effect of pH on the decomposition rate of [Cu(**2**)]^2+^ and [Cu(**3**)]^2+^ in water at 40 °C; Scheme S1: Hypothesized mechanism for the decomposition of [Cu(azacyclam)]^2+^ complexes in water displaying the nucleophilic behavior of OH−; Figure S14: Decomposition of [Cu(**2**)]^2+^ and [Cu(**3**)]^2+^ in the presence of different anions; Figure S15: ESI-MS spectra of aqueous solutions of the investigated complexes after decomposition with excess sulfite; Figure S16: Absorption spectrum in the visible range of an aqueous solution of [Cu(**1**)]^2+^ at pH 7.2 before decomposition and after decomposition in the absence and in the presence of Na_2_SO_3_; Table S2: Fitting data corresponding to dIF/dt vs. [SO_3_^2−^] for complexes [Cu(**2**)]^2+^ and [Cu(**3**)]^2+^; Figure S17: Emission spectra collected during the analysis of a white-wine sample by using [Cu(**2**)]^2+^ as chemical probe for sulfite and the resulting fluorescence intensity vs. time plot; Figure S18: Emission spectra collected during the analysis of a white-wine sample by using [Cu(**3**)]^2+^ as chemical probe for sulfite and the resulting fluorescence intensity vs. time plot.

## Sample Availability

Samples of the compounds are not available from the authors.

## References

[1] Fazio T., Warner C.R. (1990). A Review of Sulphites in Foods: Analytical Methodology and Reported Findings. Food Addit. Contam..

[2] Krska R., Becalski A., Braekevelt E., Koerner T., Cao X.-L., Dabeka R., Godefroy S., Lau B., Moisey J., Rawn D.F.K. (2012). Challenges and Trends in the Determination of Selected Chemical Contaminants and Allergens in Food. Anal. Bioanal. Chem..

[3] Vally H., Misso N.L. (2012). Adverse Reactions to the Sulphite Additives. Gastroenterol. Hepatol. Bed Bench.

[4] Heinzel M.A., Trüper H.G. (1978). Sulfite Formation by Wine Yeasts. Arch. Microbiol..

[5] Stine C.J., Boatright W.L., Lu G. (2004). Intrinsic Sulfite Content of Isolated Soy Proteins. J. Am. Oil Chem. Soc..

[6] Vally H., Misso N.L.A., Madan V. (2009). Clinical Effects of Sulphite Additives. Clin. Exp. Allergy.

[7] Lester M.R. (1995). Sulfite Sensitivity: Significance in Human Health. J. Am. Coll. Nutr..

[8] U.S. Food and Drug Administration Code of Federal Regulations, 21CFR130.9. https://www.accessdata.fda.gov/scripts/cdrh/cfdocs/cfcfr/cfrsearch.cfm?fr=130.9.

[9] Lawrence J.F., Chadha R.K., Ménard C. (1990). Comparison of Three Liquid Chromatographic Methods with FDA Optimized Monier-Williams Method for Determination of Total Sulfite in Foods. J. Assoc. Off. Anal. Chem..

[10] (2000). European Commission (EC) Directive 2000/13/EC on the Approximation of the Laws of the Member States Relating to the Labeling, Presentation, and Advertising of Foodstuffs; EC. https://www.fsai.ie/uploadedFiles/Consol_Dir2000_13.pdf.

[11] Lim H.-S., Park S.-K., Kim S.-H., Song S.-B., Jang S.-J., Kim M. (2014). Comparison of Four Different Methods for the Determination of Sulfites in Foods Marketed in South Korea. Food Addit. Contam. Part A.

[12] Ensafi A.A., Karimi-Maleh H., Keyvanfard M. (2013). A New Voltammetric Sensor for the Determination of Sulfite in Water and Wastewater Using Modified-Multiwall Carbon Nanotubes Paste Electrode. Int. J. Environ. Anal. Chem..

[13] Haskins J.E., Kendall H., Baird R.B. (1984). A Low Level Spectrophotometric Method for the Determination of Sulfite in Water. Water Res..

[14] Li Y., Zhao M. (2006). Simple Methods for Rapid Determination of Sulfite in Food Products. Food Control..

[15] Keil R., Hampp R., Ziegler H. (1989). Cycling Technique for the Determination of Femtomole Amounts of Sulfite. Anal. Chem..

[16] Pundir C.S., Rawal R. (2013). Determination of Sulfite with Emphasis on Biosensing Methods: A Review. Anal. Bioanal. Chem..

[17] McFeeters R.F., Barish A.O. (2003). Sulfite Analysis of Fruits and Vegetables by High-Performance Liquid Chromatography (HPLC) with Ultraviolet Spectrophotometric Detection. J. Agric. Food Chem..

[18] Robbins K.S., Shah R., MacMahon S., de Jager L.S. (2015). Development of a Liquid Chromatography-Tandem Mass Spectrometry Method for the Determination of Sulfite in Food. J. Agric. Food Chem..

[19] Kim H.-J., Kim Y.-K. (2006). Analysis of Free and Total Sulfites in Food by Ion Chromatography with Electrochemical Detection. J. Food Sci..

[20] Daunoravicius Z., Padarauskas A. (2002). Capillary Electrophoretic Determination of Thiosulfate, Sulfide and Sulfite Using in-Capillary Derivatization with Iodine. Electrophoresis.

[21] Jankovskiene G., Daunoravicius Z., Padarauskas A. (2001). Capillary Electrophoretic Determination of Sulfite Using the Zone-Passing Technique of in-Capillary Derivatization. J. Chromatogr. A.

[22] Monier-Williams G.W. (1927). Ministry of Health. The Determination of Sulphur Dioxide in Foods. Analyst.

[23] Vahl J., Converse J. (1980). Ripper Procedure for Determining Sulfur Dioxide in Wine: Collaborative Study. J.-Assoc. Off. Anal. Chem..

[24] AOAC (2019). Optimized Monier-Williams Method. Official Methods of Analysis.

[25] AOAC (2019). Sulfite (Free) in Wines. Official Methods of Analysis.

[26] AOAC (2019). Sulfite (Total) in Foods and Beverages. Official Methods of Analysis.

[27] AOAC (2019). Sulfites in Foods and Beverages. Official Methods of Analysis.

[28] Carlos K.S., Treblin M., de Jager L.S. (2019). Comparison and Optimization of Three Commercial Methods with an LC-MS/MS Method for the Determination of Sulfites in Food and Beverages. Food Chem..

[29] Gale P.A., Caltagirone C. (2018). Fluorescent and Colorimetric Sensors for Anionic Species. Coord. Chem. Rev..

[30] Aletti A.B., Gillen D.M., Gunnlaugsson T. (2018). Luminescent/Colorimetric Probes and (Chemo-) Sensors for Detecting Anions Based on Transition and Lanthanide Ion Receptor/Binding Complexes. Coord. Chem. Rev..

[31] Kim D.-S., Chung Y.-M., Jun M., Ahn K.H. (2009). Selective Colorimetric Sensing of Anions in Aqueous Media through Reversible Covalent Bonding. J. Org. Chem..

[32] You L., Zha D., Anslyn E.V. (2015). Recent Advances in Supramolecular Analytical Chemistry Using Optical Sensing. Chem. Rev..

[33] Lo Presti M., Martínez-Máñez R., Ros-Lis J.V., Batista R.M.F., Costa S.P.G., Raposo M.M.M., Sancenón F. (2018). A Dual Channel Sulphur-Containing a Macrocycle Functionalised BODIPY Probe for the Detection of Hg(Ii) in a Mixed Aqueous Solution. New J. Chem.

[34] Harvey P., Nonat A., Platas-Iglesias C., Natrajan L.S., Charbonnière L.J. (2018). Sensing Uranyl(VI) Ions by Coordination and Energy Transfer to a Luminescent Europium(III) Complex. Angew. Chem. Int. Ed..

[35] Yang Y., Zhao Q., Feng W., Li F. (2013). Luminescent Chemodosimeters for Bioimaging. Chem. Rev..

[36] Sancenón F., Pascual L., Oroval M., Aznar E., Martínez-Máñez R. (2015). Gated Silica Mesoporous Materials in Sensing Applications. ChemistryOpen.

[37] Toscani A., Marín-Hernández C., Robson J.A., Chua E., Dingwall P., White A.J.P., Sancenón F., de la Torre C., Martínez-Máñez R., Wilton-Ely J.D.E.T. (2019). Highly Sensitive and Selective Molecular Probes for Chromo-Fluorogenic Sensing of Carbon Monoxide in Air, Aqueous Solution and Cells. Chem. Eur. J..

[38] Cotruvo J.A., Aron A.T., Ramos-Torres K.M., Chang C.J. (2015). Synthetic Fluorescent Probes for Studying Copper in Biological Systems. Chem. Soc. Rev..

[39] Yang X.-F., Zhao M., Wang G. (2011). A Rhodamine-Based Fluorescent Probe Selective for Bisulfite Anion in Aqueous Ethanol Media. Sens. Actuators B Chem..

[40] Sun Y.-Q., Wang P., Liu J., Zhang J., Guo W. (2012). A Fluorescent Turn-on Probe for Bisulfite Based on Hydrogen Bond-Inhibited C—N Isomerization Mechanism. Analyst.

[41] Cheng X., Jia H., Feng J., Qin J., Li Z. (2013). “Reactive” Probe for Hydrogen Sulfite: Good Ratiometric Response and Bioimaging Application. Sens. Actuators B Chem..

[42] Yin C., Li X., Yue Y., Chao J., Zhang Y., Huo F. (2017). A New Fluorescent Material and Its Application in Sulfite and Bisulfite Bioimaging. Sens. Actuators B Chem..

[43] Sun Y.-Q., Liu J., Zhang J., Yang T., Guo W. (2013). Fluorescent Probe for Biological Gas SO2 Derivatives Bisulfite and Sulfite. Chem. Commun..

[44] Zhou F., Feng H., Li H., Wang Y., Zhang Z., Kang W., Jia H., Yang X., Meng Q., Zhang R. (2020). Red-Emission Probe for Ratiometric Fluorescent Detection of Bisulfite and Its Application in Live Animals and Food Samples. ACS Omega.

[45] Wu M.-Y., He T., Li K., Wu M.-B., Huang Z., Yu X.-Q. (2013). A Real-Time Colorimetric and Ratiometric Fluorescent Probe for Sulfite. Analyst.

[46] Santos-Figueroa L.E., Giménez C., Agostini A., Aznar E., Marcos M.D., Sancenón F., Martínez-Máñez R., Amorós P. (2013). Selective and Sensitive Chromofluorogenic Detection of the Sulfite Anion in Water Using Hydrophobic Hybrid Organic–Inorganic Silica Nanoparticles. Angew. Chem..

[47] Xu G., Wu H., Liu X., Feng R., Liu Z. (2015). A Simple Pyrene-Pyridinium-Based Fluorescent Probe for Colorimetric and Ratiometric Sensing of Sulfite. Dyes Pigment..

[48] Zhang Q., Zhang Y., Ding S., Zhang H., Feng G. (2015). A Near-Infrared Fluorescent Probe for Rapid, Colorimetric and Ratiometric Detection of Bisulfite in Food, Serum, and Living Cells. Sens. Actuators B Chem..

[49] Tan L., Lin W., Zhu S., Yuan L., Zheng K. (2014). A Coumarin-Quinolinium-Based Fluorescent Probe for Ratiometric Sensing of Sulfite in Living Cells. Org. Biomol. Chem..

[50] Choi M.G., Hwang J., Eor S., Chang S.-K. (2010). Chromogenic and Fluorogenic Signaling of Sulfite by Selective Deprotection of Resorufin Levulinate. Org. Lett..

[51] Gu X., Liu C., Zhu Y.-C., Zhu Y.-Z. (2011). A Boron-Dipyrromethene-Based Fluorescent Probe for Colorimetric and Ratiometric Detection of Sulfite. J. Agric. Food Chem..

[52] Chen S., Hou P., Wang J., Song X. (2012). A Highly Sulfite-Selective Ratiometric Fluorescent Probe Based on ESIPT. RSC Adv..

[53] Ma X., Liu C., Shan Q., Wei G., Wei D., Du Y. (2013). A Fluorescein-Based Probe with High Selectivity and Sensitivity for Sulfite Detection in Aqueous Solution. Sens. Actuators B Chem..

[54] Fabbrizzi L., Licchelli M., Mosca L., Poggi A. (2010). Template Synthesis of Azacyclam Metal Complexes Using Primary Amides as Locking Fragments. Coord. Chem. Rev..

[55] Abbà F., De Santis G., Fabbrizzi L., Licchelli M., Manotti Lanfredi A.M., Pallavicini P., Poggi A., Ugozzoli F. (1994). Nickel(II) Complexes of Azacyclams: Oxidation and Reduction Behavior and Catalytic Effects in the Electroreduction of Carbon Dioxide. Inorg. Chem..

[56] De Blas A., De Santis G., Fabbrizzi L., Licchelli M., Pallavicini P. (1993). Novel Routes to Functionalized Cyclam-like Macrocycles. Pure Appl. Chem..

[57] Tsymbal L.V., Andriichuk I.L., Shova S., Trzybiński D., Woźniak K., Arion V.B., Lampeka Y.D. (2021). Coordination Polymers of the Macrocyclic Nickel(II) and Copper(II) Complexes with Isomeric Benzenedicarboxylates: The Case of Spatial Complementarity between the Bis-Macrocyclic Complexes and o-Phthalate. Cryst. Growth Des..

[58] Lee J.H., Moon H.R. (2018). Structural Diversity of Metal–Organic Frameworks via Employment of Azamacrocycles as a Building Block. J. Incl. Phenom. Macrocycl. Chem..

[59] Suh M.P., Ko J.W., Choi H.J. (2002). A Metal−Organic Bilayer Open Framework with a Dynamic Component: Single-Crystal-to-Single-Crystal Transformations. J. Am. Chem. Soc..

[60] Ju P., Jiang L., Lu T.-B. (2013). An Unprecedented Dynamic Porous Metal–Organic Framework Assembled from Fivefold Interlocked Closed Nanotubes with Selective Gas Adsorption Behaviors. Chem Commun.

[61] Stackhouse C.A., Ma S. (2018). Azamacrocyclic-Based Metal Organic Frameworks: Design Strategies and Applications. Polyhedron.

[62] Savastano M., Monini V., Bazzicalupi C., Bianchi A. (2022). Bidimensional Polyiodide Netting Stabilized by a Cu(II) Macrocyclic Complex. Inorganics.

[63] Martínez-Camarena Á., Savastano M., Blasco S., Delgado-Pinar E., Giorgi C., Bianchi A., García-España E., Bazzicalupi C. (2022). Assembly of Polyiodide Networks with Cu(II) Complexes of Pyridinol-Based Tetraaza Macrocycles. Inorg. Chem..

[64] Liu D.-C., Zhong D.-C., Lu T.-B. (2020). Non-Noble Metal-Based Molecular Complexes for CO_2_ Reduction: From the Ligand Design Perspective. EnergyChem.

[65] Wang J.-W., Zhong D.-C., Lu T.-B. (2018). Artificial Photosynthesis: Catalytic Water Oxidation and CO_2_ Reduction by Dinuclear Non-Noble-Metal Molecular Catalysts. Coord. Chem. Rev..

[66] Sayed S.E., Licchelli M., Martínez-Máñez R., Sancenón F. (2017). Capped Mesoporous Silica Nanoparticles for the Selective and Sensitive Detection of Cyanide. Chem. Asian J..

[67] El Sayed S., Milani M., Licchelli M., Martínez-Máñez R., Sancenón F. (2015). Hexametaphosphate-Capped Silica Mesoporous Nanoparticles Containing Cu^II^ Complexes for the Selective and Sensitive Optical Detection of Hydrogen Sulfide in Water. Chem.-Eur. J..

[68] Fabbrizzi L., Licchelli M., Manotti Lanfredi A.M., Vassalli O., Ugozzoli F. (1996). Template Synthesis of a Tetraaza Macrocycle Which Involves Benzaldehyde Rather Than Formaldehyde as a Building Block. Isolation and Structure Determination of the Open-Chain Schiff Base Intermediate Complex. Inorg. Chem..

[69] Fabbrizzi L., Lanfredi A.M.M., Pallavicini P., Perotti A., Taglietti A., Ugozzoli F. (1991). Crystal and Molecular Structure and Solution Behaviour of Low-Spin (3-Methyl-1,3,5,8,12-Pentaazacyclotetradecane)Κ4N1,N5,N8,N12)Nickel(II) Diperchlorate. J. Chem Soc. Dalton Trans..

[70] Rosokha S.V., Lampeka Y.D. (1991). A New One-Pot Synthesis of Bis(Macrocyclic) Complexes: Preparation and Characterization of Nickel Complexes with Bis(Pentaazamacrocyclic) Ligands. J. Chem. Soc. Chem. Commun..

[71] Hay R.W., Danby A., Lightfoot P., Lampeka Y.D. (1997). Padlock Macrocyclic Complexes. The Synthesis of a Range of Nickel(II) Complexes of N-Alkyl Azacyclams and the Crystal Structure of (3-Ethyl-1,3,5,8,12-Penta-Azacyclotetradecane) Nickel(II) Perchlorate. Polyhedron.

[72] Vengatajalabathy Gobi K., Ohsaka T. (1998). Electrochemical and Spectral Properties of Novel Dinickel(II) and Dicopper(II) Complexes with N,N-Linked Bis(Pentaazacyclotetradecane). Electrochim. Acta.

[73] Bernhardt P.V., Hayes E.J. (1998). Aminotriazines as Locking Fragments in Macrocyclic Synthesis. Inorg. Chem..

[74] De Blas A., De Santis G., Fabbrizzi L., Licchelli M., Lanfredi A.M.M., Morosini P., Pallavicini P., Ugozzoli F. (1993). Amides and Sulfonamides: Efficient Molecular Padlocks for the Template Synthesis of Azacyclam (1,3,5,8,12-Pentaazacyclotetradecane) Macrocycles. J. Chem. Soc. Dalton Trans..

[75] De Santis G., Fabbrizzi L., Licchelli M., Mangano C., Pallavicini P. (1993). The Copper(I) Complex of a Metallocyclam-Functionalized Phenanthroline: A Poorly Stable Species That Is Very Resistant to Oxidation. Inorg. Chem..

[76] De Blas A., De Santis G., Fabbrizzi L., Licchelli M., Lanfredi A.M.M., Pallavicini P., Poggi A., Ugozzoli F. (1993). Pyridines with an Appended Metallocyclam Subunit. Versatile Building Blocks to Supramolecular Multielectron Redox Systems. Inorg. Chem..

[77] De Santis G., Fabbrizzi L., Licchelli M., Mangano C., Pallavicini P., Poggi A. (1993). Ferrocene-Metallocyclam Conjugates: New Redox Systems Whose Two-Electron Activity Can Be Modulated through the Medium. Inorg. Chem..

[78] Fabbrizzi L., Licchelli M., De Santis G., Sardone N., Velders A.H. (1996). Fluorescence Redox Switching Systems Operating through Metal Centres: The Ni^III^/Ni^II^ Couple. Chem. Eur. J..

[79] Boiocchi M., Fabbrizzi L., Garolfi M., Licchelli M., Mosca L., Zanini C. (2009). Templated Synthesis of Copper(II) Azacyclam Complexes Using Urea as a Locking Fragment and Their Metal-Enhanced Binding Tendencies towards Anions. Chem. Eur. J..

[80] Amendola V., Fabbrizzi L., Foti F., Licchelli M., Mangano C., Pallavicini P., Poggi A., Sacchi D., Taglietti A. (2006). Light-Emitting Molecular Devices Based on Transition Metals. Coord. Chem. Rev..

[81] Boiocchi M., Fabbrizzi L., Licchelli M., Sacchi D., Vázquez M., Zampa C. (2003). A Two-Channel Molecular Dosimeter for the Optical Detection of Copper (II). Chem. Commun..

[82] Sasakura K., Hanaoka K., Shibuya N., Mikami Y., Kimura Y., Komatsu T., Ueno T., Terai T., Kimura H., Nagano T. (2011). Development of a Highly Selective Fluorescence Probe for Hydrogen Sulfide. J. Am. Chem. Soc..

[83] Santos-Figueroa L.E., de la Torre C., El Sayed S., Sancenón F., Martínez-Máñez R., Costero A.M., Gil S., Parra M. (2014). Highly Selective Fluorescence Detection of Hydrogen Sulfide by Using an Anthracene-Functionalized Cyclam–Cu^II^ Complex. Eur. J. Inorg. Chem..

[84] Mirra S., Strianese M., Pellecchia C. (2017). A Cyclam-Based Fluorescent Ligand as a Molecular Beacon for Cu^2+^ and H_2_S Detection. Eur. J. Inorg. Chem..

[85] Hathaway B.J. (1984). A new look at the stereochemistry and electronic properties of complexes of the copper(II) ion. Structure and Bonding.

[86] Licchelli M., Milani M., Pizzo S., Poggi A., Sacchi D., Boiocchi M. (2012). Synthesis of Novel Diazacyclam Copper(II) Complexes by Template Reaction Involving Sulphonamides as Locking Fragments. Inorg. Chim. Acta.

[87] Thoem V.J., Boeyens J.C.A., McDougall G.J., Hancock R.D. (1984). Origin of the High Ligand Field Strength and Macrocyclic Enthalpy in Complexes of Nitrogen-Donor Macrocycles. J. Am. Chem. Soc..

[88] Groom C.R., Bruno I.J., Lightfoot M.P., Ward S.C. (2016). The Cambridge Structural Database. Acta Crystallogr. Sect. B.

[89] Cabbiness D.K., Margerum D.W. (1970). Effect of Macrocyclic Structures on the Rate of Formation and Dissociation of Copper(II) Complexes. J. Am. Chem. Soc..

[90] Busch D.H. (1978). Distinctive Coordination Chemistry and Biological Significance of Complexes with Macrocyclic Ligands. Acc. Chem. Res..

[91] Mansour A.M. (2014). Crystal Structure, DFT, Spectroscopic and Biological Activity Evaluation of Analgin Complexes with Co(Ii), Ni(II) and Cu(II). Dalton Trans..

[92] Borman P., Elder D. (2017). Q2(R1) Validation of Analytical Procedures. ICH Qual. Guidel..

[93] Savitzky A., Golay M.J.E. (1964). Smoothing and Differentiation of Data by Simplified Least Squares Procedures. Anal. Chem..

[94] Virtanen P., Gommers R., Oliphant T.E., Haberland M., Reddy T., Cournapeau D., Burovski E., Peterson P., Weckesser W., Bright J. (2020). SciPy 1.0: Fundamental Algorithms for Scientific Computing in Python. Nat. Methods.

[95] Hunter J.D. (2007). Matplotlib: A 2D Graphics Environment. Comput. Sci. Eng..

[96] van der Walt S., Colbert S.C., Varoquaux G. (2011). The NumPy Array: A Structure for Efficient Numerical Computation. Comput. Sci. Eng..

[97] Perez F., Granger B.E. (2007). IPython: A System for Interactive Scientific Computing. Comput. Sci. Eng..

[98] Newville M., Otten R., Nelson A., Ingargiola A., Stensitzki T., Allan D., Fox A., Carter F., Michał, Pustakhod D. Lmfit/Lmfit-Py 0.9.13.

[99] Farrugia L.J. (2012). WinGX and It ORTEP for Windows: An Update. J. Appl. Crystallogr..

[100] North A.C.T., Phillips D.C., Mathews F.S. (1968). A Semi-Empirical Method of Absorption Correction. Acta Crystallogr. Sect. A.

[101] Brucker (2003). SAINT Software Reference Manual, Version 6.

[102] Krause L., Herbst-Irmer R., Sheldrick G.M., Stalke D. (2015). Comparison of Silver and Molybdenum Microfocus X-Ray Sources for Single-Crystal Structure Determination. J. Appl. Crystallogr..

[103] Altomare A., Burla M.C., Camalli M., Cascarano G.L., Giacovazzo C., Guagliardi A., Moliterni A.G., Polidori G., Spagna R. (1999). SIR97: A New Tool for Crystal Structure Determination and Refinement. J. Appl. Crystallogr..

[104] Sheldrick G.M. (2015). Crystal Structure Refinement with SHELXL. Acta Crystallogr. Sect. C Struct. Chem..

