# Supplementary material for: Fluorogenic Detection of Sulfite in Water by Using Copper(II) Azacyclam Complexes
Source: Molecules. 2022 Mar 12;27(6):1852. doi: 10.3390/molecules27061852 (PMC8951044; doi:10.3390/molecules27061852)
Supplement: Supplementary file 1 [file molecules-27-01852-s001.zip › molecules-1614301-supplementary.pdf]

FLUOROGENIC DETECTION OF SULFITE IN WATER BY USING A COPPER(II) AZACYCLAM COMPLEXES

Carlo Ciarrocchi, Donatella Sacchi, Massimo Boiocchi, Maduka L. Weththimuni, Alessio Orbelli Biroli, Maurizio Licchelli

SUPPLEMENTARY INFORMATION

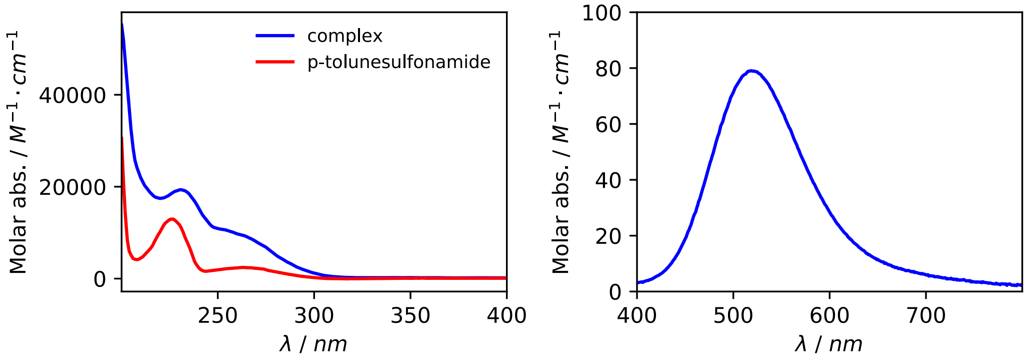

Figure S1. Absorption spectra of complex $[Cu(\mathbf{1})]^{2+}$ in aqueous solution 2×10^{-5} M (left) and 2×10^{-4} M (right). Spectrum of p-toluensulfonamide is also reported (left) for comparison.

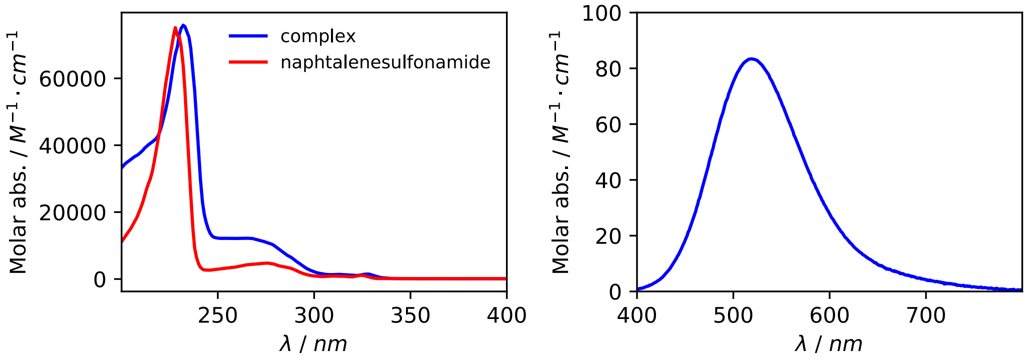

Figure S2. Absorption spectra of complex $[Cu(\mathbf{2})]^{2+}$ in aqueous solution 2×10^{-5} M (left) and 2×10^{-4} M (right). Spectrum of 2-naphthalensulfonamide is also reported (left) for comparison.

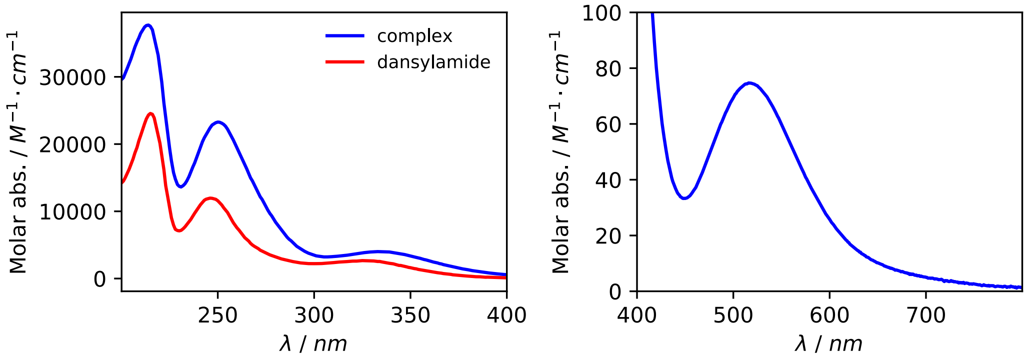

Figure S3. Absorption spectra of complex $[Cu(\mathbf{3})]^{2+}$ in aqueous solution 2×10^{-5} M (left) and 2×10^{-4} M (right). Spectrum of dansylamide is also reported (left) for comparison.

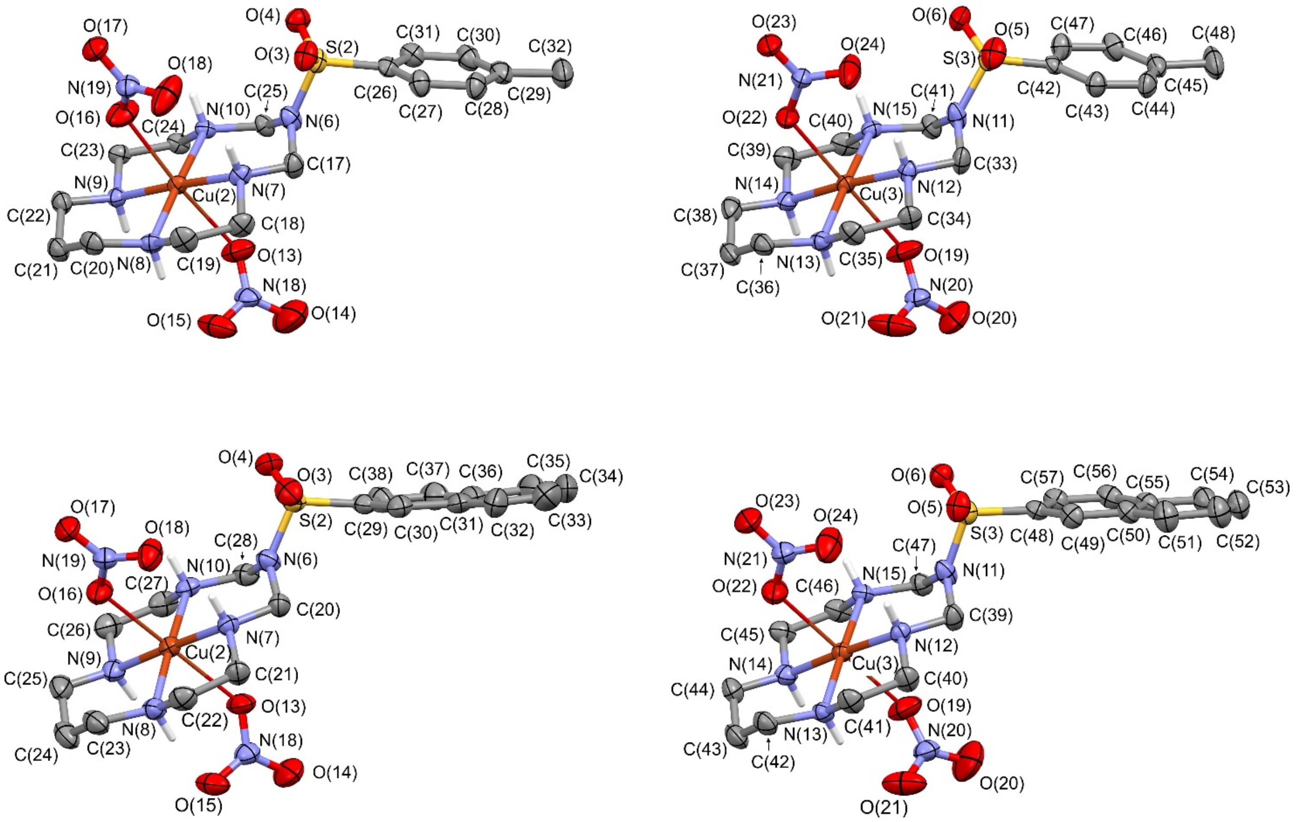

Figure S4. Plot showing thermal ellipsoids for the other independent azacyclam ligands occurring in the 3[[Cu(**1**)](NO_3)$_2$] (top) and 3[[Cu(**2**)](NO_3)$_2$]·2H_2O] (bottom) crystals (ellipsoids are drawn at the 30% probability level; only H bonded to the secondary amines are shown). Weak axial Cu-O bond interactions are drawn as thin sticks.

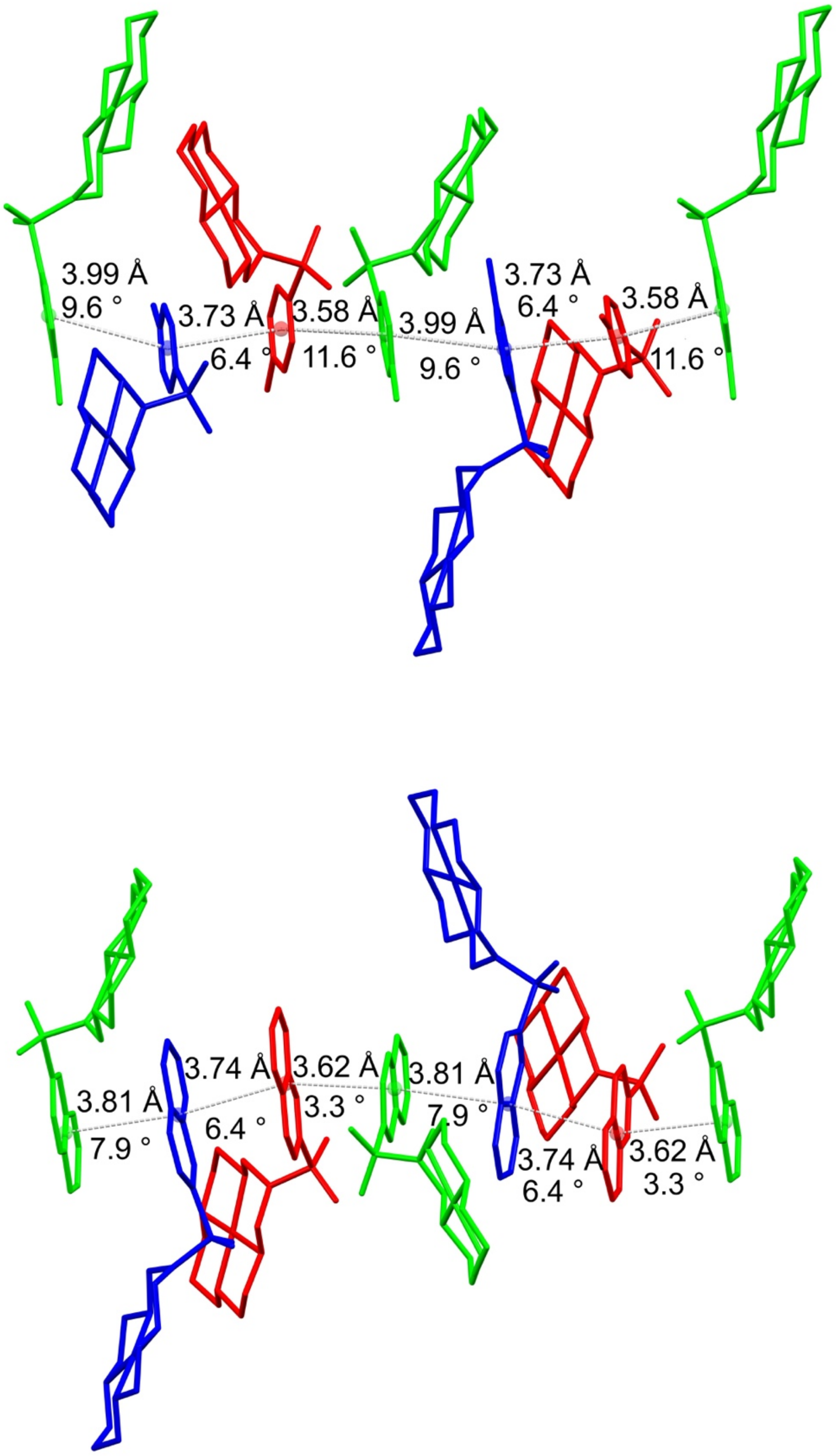

Figure S5. Simplified sketches of the supramolecular chains maintained by face-to-face π-stacking interactions in the 3[[Cu(**1**)](NO_3)$_2$] (top) and 3[[Cu(**2**)](NO_3)$_2$]·2H_2O] (bottom) crystals (H-atoms, nitrate counterions and water solvent molecules are omitted for clarity; symmetrically equivalent molecules have the same colour). The numerical values refer to the centroid-centroid separations (drawn as dashed lines) and to the dihedral angles between facing aromatic rings.

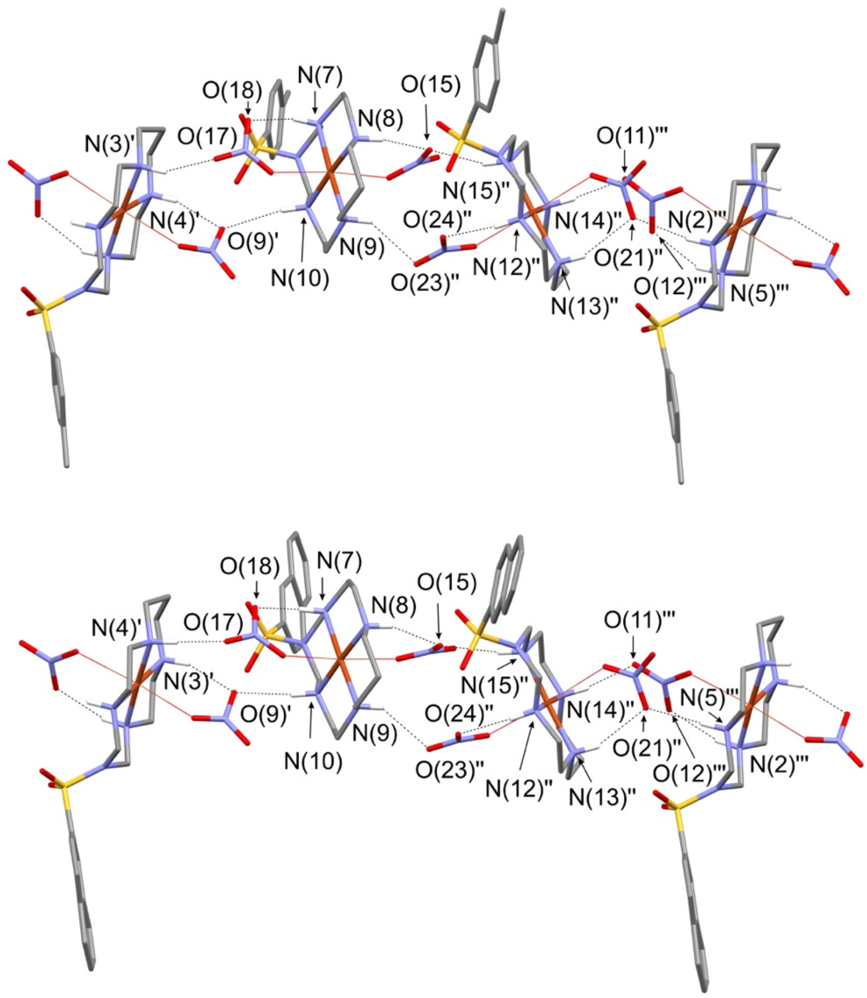

Figure S6. Simplified sketches of the N-H···O H-bonds in the 3[[Cu(**1**)](NO_3)$_2$] (top) and 3[[Cu(**2**)](NO_3)$_2$]·$2H_2O$] (bottom) crystals (only H-atoms involved in H-bonds are drawn, water solvent molecules are omitted for clarity). Atom names identify the independent N-H···O interactions, drawn as dashed lines. Symmetry code: for top (') = x, $^{1}/_{2}$-y, -$^{1}/_{2}$+z; ('') = 1-x, $^{1}/_{2}$+y, $^{1}/_{2}$-z; (''') = x, $^{3}/_{2}$-y, -$^{1}/_{2}$+z; for bottom (') = $^{1}/_{2}$+x, $^{1}/_{2}$-y, -z ; ('') = 1-x, $^{1}/_{2}$+y, $^{1}/_{2}$-z; (''') = $^{1}/_{2}$+x, $^{3}/_{2}$-y, -z.

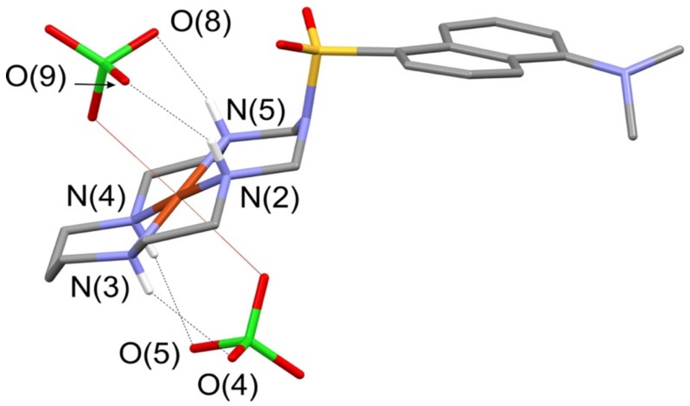

Figure S7. A simplified sketch of the N-H···O H-bonds occurring in the [Cu(**3**)](ClO_4)$_2$ crystal (only H-atoms involved in H-bonds are drawn). Atom names are reported only for N and O species involved in H-bonds, drawn as dashed lines.

Table S1. Geometrical features of the N-H···O H-bonds occurring in the studied crystals. Atom names refer to the atom positions shown in Figure S6 and S7.

D donor group	D···A [Å]	H···A [Å]	D-H···A [°]	A acceptor atom
		3[[Cu(**1**)]($(NO_3)_2$)]		
N(2)'''-H(2N)'''	3.051(6)	2.21(2)	155(4)	O(21)''
N(3)'-H(3N)'	2.949(5)	2.13(3)	152(4)	O(17)
N(4)'-H(4N)'	2.989(6)	2.13(2)	161(4)	O(9)'
N(5)'''-H(5N)'''	2.975(6)	2.14(2)	154(4)	O(12)'''
N(7)-H(7N)	3.044(6)	2.21(2)	154(4)	O(18)
N(8)-H(8N)	3.106(6)	2.30(3)	150(4)	O(15)
N(9)-H(9N)	3.091(5)	2.32(3)	144(4)	O(23)''
N(10)-H(10N)	3.158(5)	2.28(2)	168(4)	O(9)'
N(12)''-H(12N)''	2.951(6)	2.11(2)	156(4)	O(24)''
N(13)''-H(13N)''	3.166(7)	2.34(2)	153(4)	O(21)''
N(14)''-H(14N)''	3.066(6)	2.26(3)	150(4)	O(11)'''
N(15)''-H(15N)''	2.994(6)	2.15(2)	157(4)	O(15)
Symmetry code: (') = x, $^1/_2$-y, -$^1/_2$+z; ('') = 1-x, $^1/_2$+y, $^1/_2$-z; (''') = x, $^3/_2$-y, -$^1/_2$+z				
		3[[Cu(**2**)]($(NO_3)_2$)]·$2H_2O$]		
N(2)'''-H(2N)'''	2.980(7)	2.11(2)	165(5)	O(12)'''
N(3)'-H(3N)'	3.008(7)	2.18(3)	154(5)	O(9)'
N(4)'-H(4N)'	3.042(6)	2.21(2)	155(4)	O(17)
N(5)'''-H(5N)'''	3.058(6)	2.19(2)	163(4)	O(21)''
N(7)-H(7N)	2.975(6)	2.12(2)	158(5)	O(18)
N(8)-H(8N)	3.013(6)	2.17(3)	156(5)	O(15)
N(9)-H(9N)	3.038(7)	2.30(4)	140(5)	O(23)''
N(10)-H(10N)	2.993(6)	2.10(2)	170(5)	O(9)'
N(12)''-H(12N)''	2.998(7)	2.16(2)	156(4)	O(24)''
N(13)''-H(13N)''	3.150(7)	2.31(2)	155(5)	O(21)''
N(14)''-H(14N)''	3.014(6)	2.23(3)	145(5)	O(11)'''
N(15)''-H(15N)''	3.094(6)	2.21(2)	172(5)	O(15)
Symmetry code: (') = $^1/_2$+x, $^1/_2$-y, -z ; ('') = 1-x, $^1/_2$+y, $^1/_2$-z; (''') = $^1/_2$+x, $^3/_2$-y, -z.				
		[Cu(**3**)]($(ClO_4)_2$)		
N(2)-H(2N)	3.100(3)	2.31(2)	147(2)	O(9)
N(3)-H(3N)	3.151(3)	2.33(2)	155(2)	O(4)
N(4)-H(4N)	3.152(3)	2.35(2)	151(2)	O(5)
N(5)-H(5N)	3.237(3)	2.47(2)	145(2)	O(8)

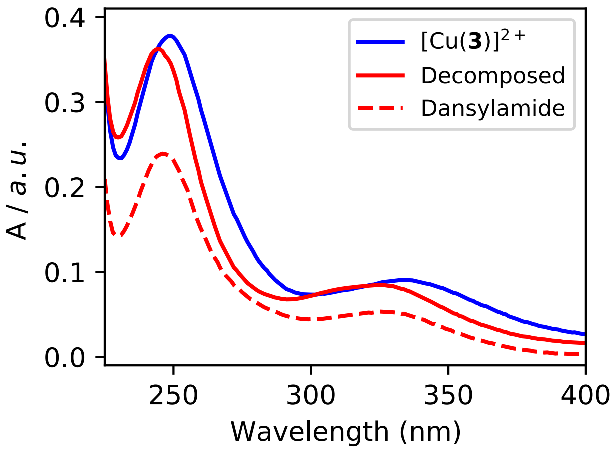

Figure S8. Absorption spectra of complex $[Cu(\mathbf{3})]^{2+}$ in aqueous solution (2×10^{-5} M) before and after decomposition obtained by heating (1 h, 70°C, pH=7.2). Spectrum of dansylamide is also reported for comparison.

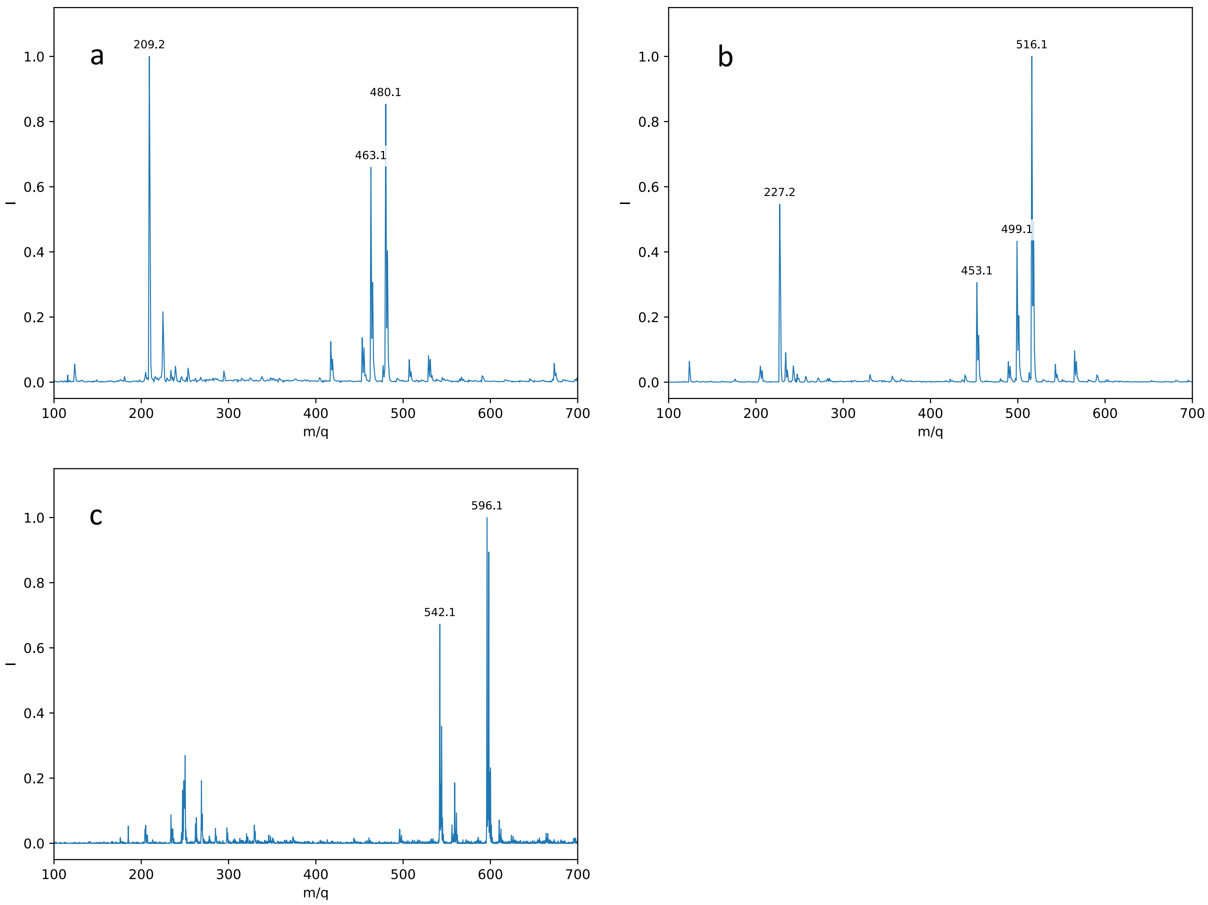

Figure S9. ESI-MS spectra of aqueous solutions of (a) $[Cu(\mathbf{1})](NO_3)_2$ (m/z = 209, $\{M\}^{2+}$; 463, $\{M+HCOO\}^{+}$; 480, $\{M+NO_3\}^{+}$; M = $[Cu(\mathbf{1})]^{2+}$) (b) $[Cu(\mathbf{2})](NO_3)_2$, (m/z = 227, $\{M\}^{2+}$; 453, $\{M\text{-}H\}^{+}$; 499, $\{M+HCOO\}^{+}$; 516, $\{M+NO_3\}^{+}$; M = $[Cu(\mathbf{2})]^{2+}$), and (c) $[Cu(\mathbf{3})](ClO_4)_2$ (m/z = 542, $\{M+HCOO\}^{+}$; 596, $\{M+ClO_4\}^{+}$; M = $[Cu(\mathbf{3})]^{2+}$).

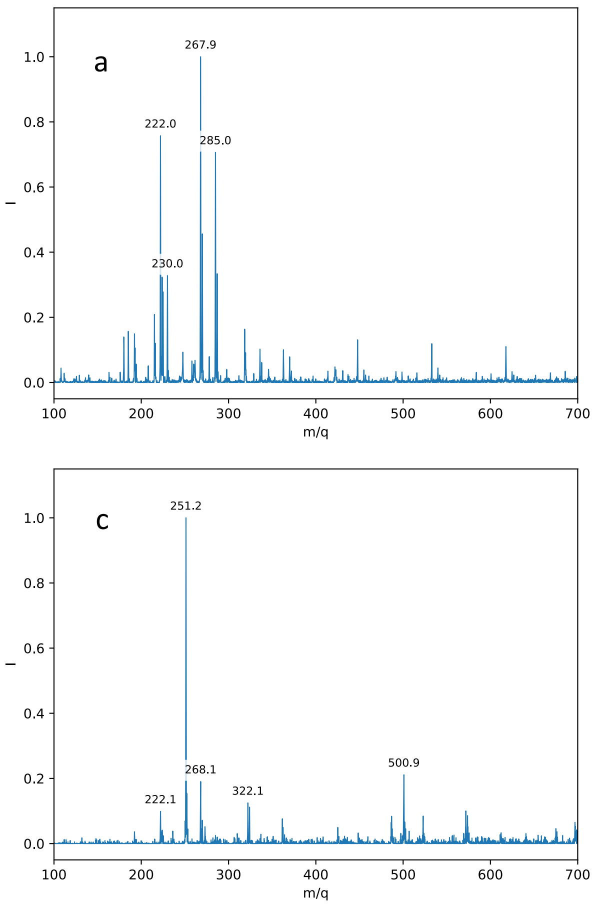

Figure S10. ESI-MS spectra of aqueous solutions of the investigated copper(II) azacyclam complexes after 1h heating at 70 °C.

(a) [Cu(**1**)](NO_3)$_2$ (m/z = 194, {*p*-toluensulfonamide+Na}$^+$; 222, {M-H}$^+$; 268, {M+HCOO}$^+$; 285 {M+NO_3}$^+$; M = [Cu(2.3.2-tet)]$^{2+}$).

(b) [Cu(**2**)](NO_3)$_2$ (m/z = 222, {M-H}$^+$; 230, {2-naphthalenesulfonamide+Na}$^+$; 268, {M+HCOO}$^+$; 285 {M+NO_3}$^+$; M = [Cu(2.3.2-tet)]$^{2+}$).

(c) [Cu(**3**)](ClO_4)$_2$ (m/z = 222, {M-H}$^+$; 251, {dansylamide+H}$^+$; 268, {M+HCOO}$^+$; 322, {M+ClO_4}$^+$; 501, {2dansylamide+H}$^+$; M = [Cu(2.3.2-tet)]$^{2+}$).

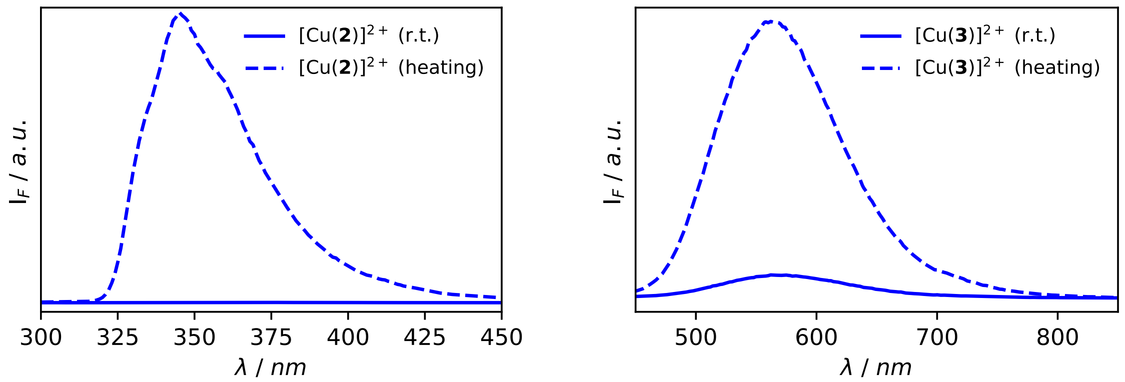

Figure S11. Emission spectra of aqueous solutions (2 x 10^{-5} M) of [Cu(**2**)]$^{2+}$ (left) and [Cu(**3**)]$^{2+}$ (right) taken at room temperature and after 1h heating at 70 °C (λ_{exc} = 275 and 325 nm, respectively)

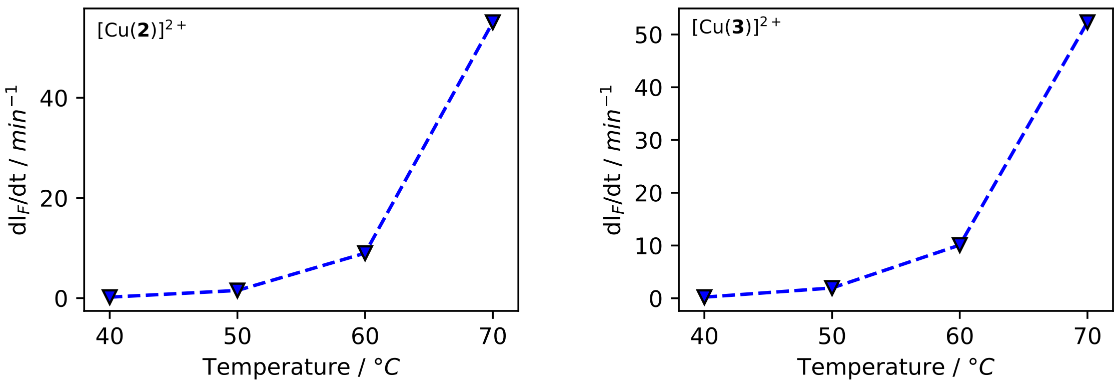

Figure S12. Effect of temperature on the decomposition rate of $[Cu(\mathbf{2})]^{2+}$ (left) and $[Cu(\mathbf{3})]^{2+}$ (right) in water at pH 7.2.

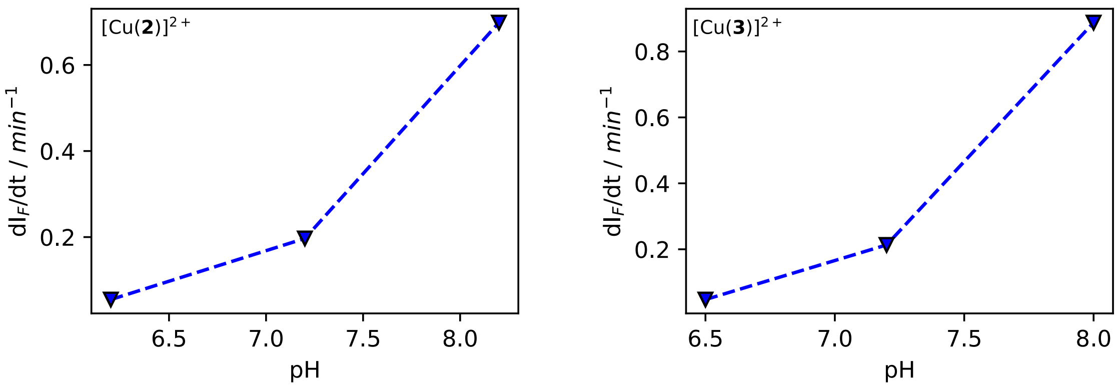

Figure S13. Effect of pH on the decomposition rate of $[Cu(\mathbf{2})]^{2+}$ (left) and $[Cu(\mathbf{3})]^{2+}$ (right) in water at 40°C.

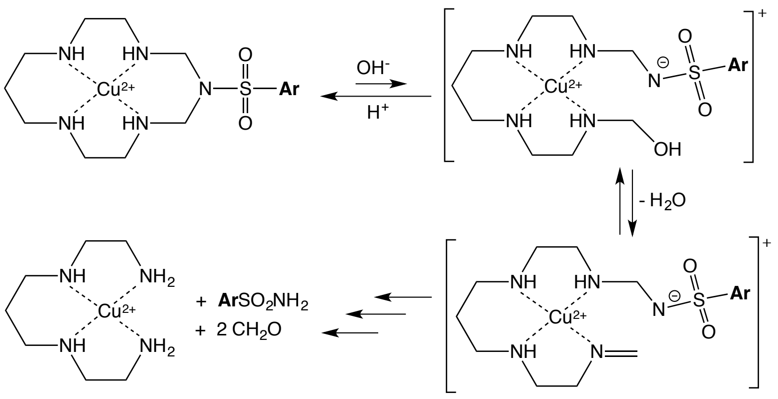

Scheme S1. Hypothesized mechanism for the decomposition of $[Cu(azacyclam)]^{2+}$ complexes in water displaying the nucleophilic behaviour of OH^{-}.

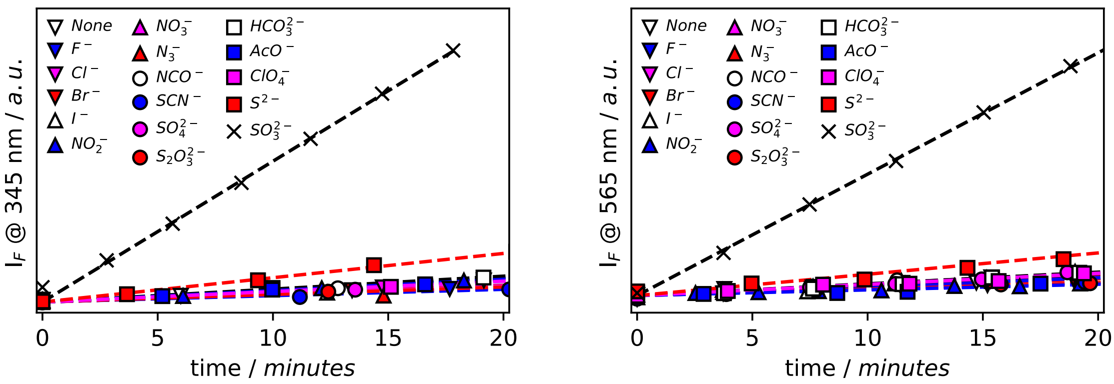

Figure S14. Decomposition of [Cu(**2**)]$^{2+}$ (left) and [Cu(**3**)]$^{2+}$ (right) 2×10^{-5} M in the presence of different anions. I_F monitoring has been limited to the first 20 min.

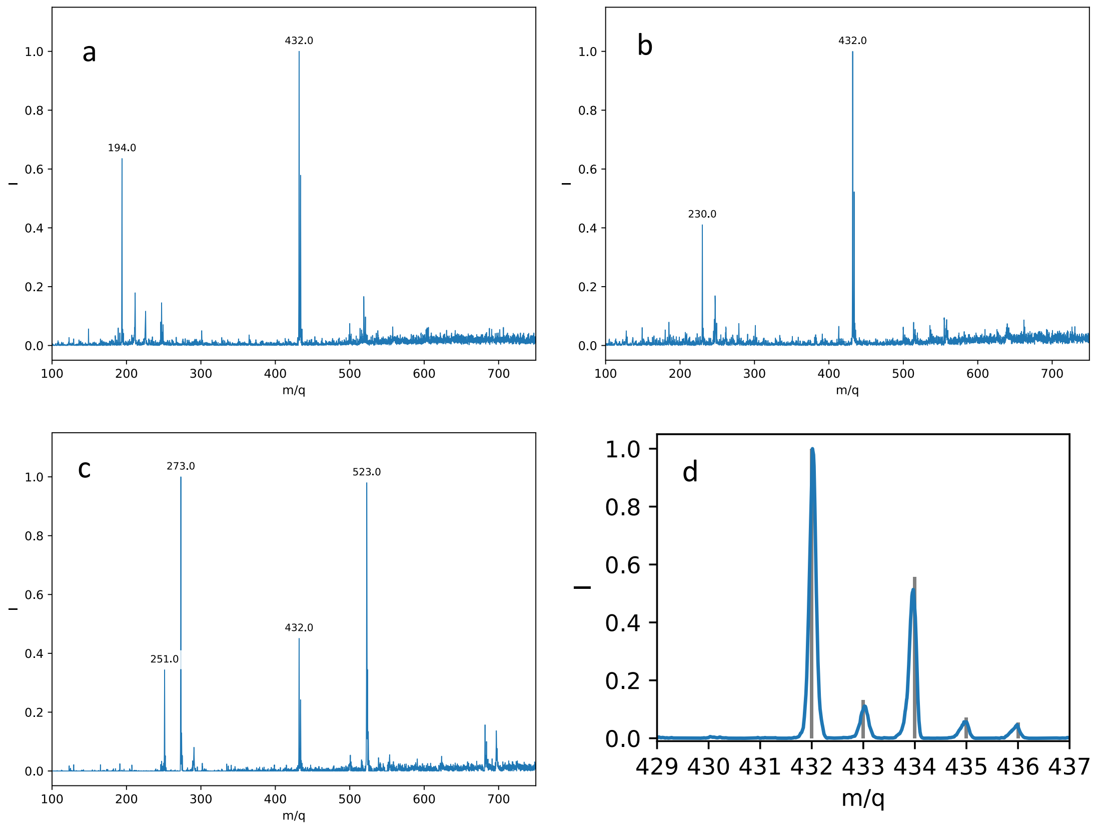

Figure S15. ESI-MS spectra of aqueous solutions of the investigated copper(II) azacyclam complexes (2.6×10^{-3} M) after decomposition with excess sulfite (2.6×10^{-2} M).

(a) [Cu(**1**)](NO_3)$_2$ (m/z = 194, {*p*-toluensulfonamide+Na}$^+$; 432, {[Cu(**4**)]^0+Na}$^+$.

(b) [Cu(**2**)](NO_3)$_2$ (m/z = 230, {2-naphthalenesulfonamide+Na}$^+$; 432, {[Cu(**4**)]^0+Na}$^+$.

(c) [Cu(**3**)](ClO_4)$_2$ (m/z = 251, {dansylamide+H}$^+$; 273, {dansylamide+Na}$^+$; 432, {[Cu(**4**)]^0+Na}$^+$. 523, {2dansylamide+Na}$^+$.

(d) Zoom scan spectrum (blue line) related to the peak at m/z 432 compared with the corresponding simulated mass spectrum (grey lines).

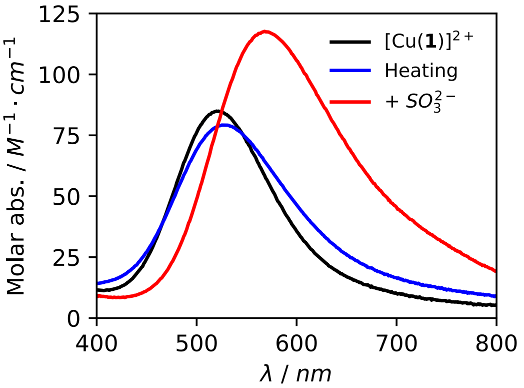

Figure S16. Absorption spectrum in the visible range of an aqueous solution of [Cu(**1**)]$^{2+}$ 2.6×10^{-3} M at pH 7.2 before decomposition (black) and after decomposition (pH 7.2, 40°C) in the absence (red) and in the presence of Na_2SO_3 (blue, 2.6×10^{-2}).

Table S2. Fitting data corresponding to dI_F/dt vs. [SO_3^{2-}] for complexes [Cu(**2**)]$^{2+}$ and [Cu(**3**)]$^{2+}$ (Standard deviation values in brackets)

	[Cu(**2**)]$^{2+}$	[Cu(**3**)]$^{2+}$
v_0	0.25(4)	0.19(9)
a	3.53(6)	3.44(12)
b	1.12(6)×10^5	1.63(14)×10^5
Plateau (v_0 + *a*)	3.78	3.63
LOD	1×10^{-6} M (0.035 ppm SO_2)	9×10^{-7} M (0.03 ppm SO_2)
LOQ	3×10^{-6} M (0.1 ppm SO_2)	2.6×10^{-6} M (0.09 ppm SO_2)

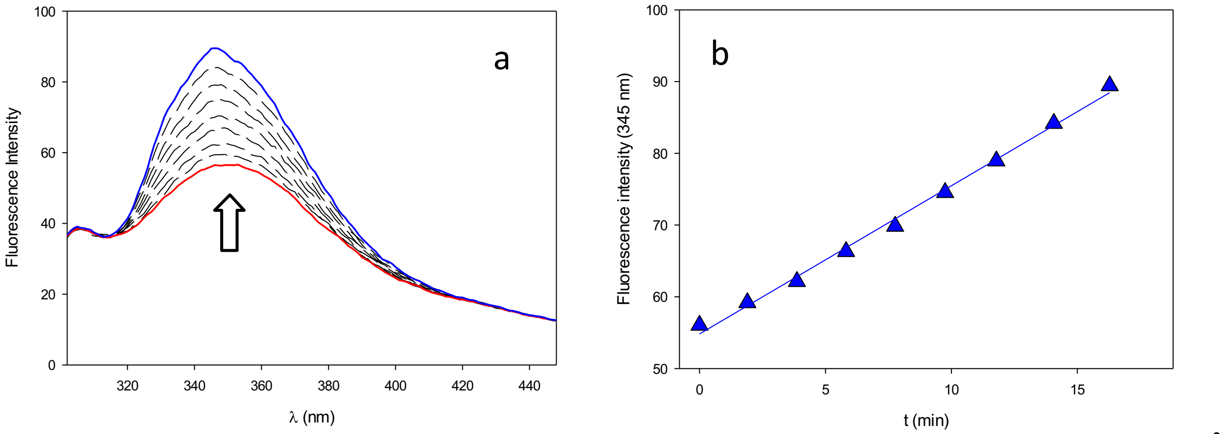

Figure S17. (a) Emission spectra collected during the analysis of a white wine sample by using [Cu(**2**)]$^{2+}$ (2×10^{-5} M; pH=7.2; T=40 °C) as chemical probe for sulfite and (b) the resulting fluorescence intensity vs. time plot.

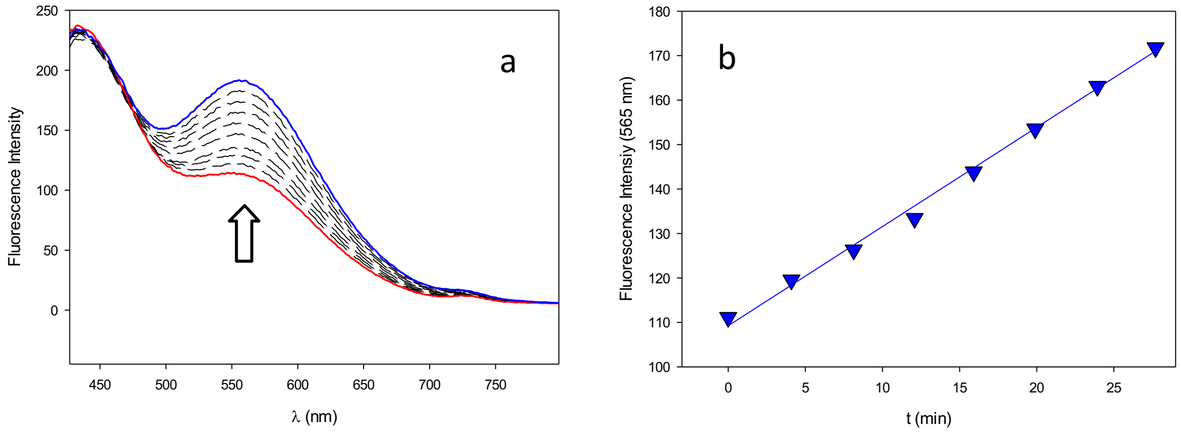

Figure S18. (a) Emission spectra collected during the analysis of a white wine sample by using [Cu(**3**)]$^{2+}$ (2×10^{-5} M; pH=7,2; T=40 °C) as chemical probe for sulfite and (b) the resulting fluorescence intensity vs. time plot.
